# NUAK1 coordinates growth factor-dependent activation of mTORC2 and Akt signaling

**DOI:** 10.1186/s13578-023-01185-2

**Published:** 2023-12-22

**Authors:** Mario Palma, Elizabeth Riffo, Alejandro Farias, Viviana Coliboro-Dannich, Luis Espinoza-Francine, Emilia Escalona, Roberto Amigo, José L. Gutiérrez, Roxana Pincheira, Ariel F. Castro

**Affiliations:** 1 Laboratorio de Transducción de Señales y Cáncer, Departamento de Bioquímica y Biología Molecular, Facultad Cs. Biológicas, Universidad de Concepción, Concepción, Chile; 2 Laboratorio de Regulación Transcripcional, Departamento de Bioquímica y Biología Molecular, Facultad Cs. Biológicas, Universidad de Concepción, Concepción, Chile

**Keywords:** Akt, Cancer signaling, Co-targeting, mTORC2, NUAK1

## Abstract

**Background:**

mTORC2 is a critical regulator of cytoskeleton organization, cell proliferation, and cancer cell survival. Activated mTORC2 induces maximal activation of Akt by phosphorylation of Ser-473, but regulation of Akt activity and signaling crosstalk upon growth factor stimulation are still unclear.

**Results:**

We identified that NUAK1 regulates growth factor-dependent activation of Akt by two mechanisms. NUAK1 interacts with mTORC2 components and regulates mTORC2-dependent activation of Akt by controlling lysosome positioning and mTOR association with this organelle. A second mechanism involves NUAK1 directly phosphorylating Akt at Ser-473. The effect of NUAK1 correlated with a growth factor-dependent activation of specific Akt substrates. NUAK1 induced the Akt-dependent phosphorylation of FOXO1/3a (Thr-24/Thr-32) but not of TSC2 (Thr-1462). According to a subcellular compartmentalization that could explain NUAK1’s differential effect on the Akt substrates, we found that NUAK1 is associated with early endosomes but not with plasma membrane, late endosomes, or lysosomes. NUAK1 was required for the Akt/FOXO1/3a axis, regulating p21CIP1, p27KIP1, and FoxM1 expression and cancer cell survival upon EGFR stimulation. Pharmacological inhibition of NUAK1 potentiated the cell death effect induced by Akt or mTOR pharmacological blockage. Analysis of human tissue data revealed that NUAK1 expression positively correlates with EGFR expression and Akt Ser-473 phosphorylation in several human cancers.

**Conclusions:**

Our results showed that NUAK1 kinase controls mTOR subcellular localization and induces Akt phosphorylation, demonstrating that NUAK1 regulates the growth factor-dependent activation of Akt signaling. Therefore, targeting NUAK1, or co-targeting it with Akt or mTOR inhibitors, may be effective in cancers with hyperactivated Akt signaling.

**Supplementary Information:**

The online version contains supplementary material available at 10.1186/s13578-023-01185-2.

## Background

The mechanistic target of rapamycin (mTOR) is an evolutionarily conserved Ser/Thr kinase that belongs to the phosphatidylinositol-3-kinase-related kinase (PIKK) family [1]. The mTOR functions are dictated by its association with different proteins, resulting in the formation of two distinct complexes: mTORC1 (mTOR complex 1) and mTORC2 (mTOR complex 2). Both complexes contain mTOR kinase, mLST8, DEPTOR, and the Tti1/Tel2 complex. Additionally, mTORC1 contains Regulatory-Associated Protein of Mammalian Target of Rapamycin (Raptor) and PRAS40. Instead, mTORC2 has Rapamycin-Insensitive Companion of mTOR (Rictor), mSIN1, and Protor ½ [2]. mTORC1 is considered a master regulator of cellular metabolism, mRNA translation, cell growth/proliferation, and migration. On the other hand, mTORC2 functions are associated with the regulation of cytoskeletal organization, cell proliferation, and cell survival. Many of these functions are in coordination with the Akt signaling [1]. Upstream mTORC1 signaling modulators have been well defined, including TSC1/2, PRAS40, Rheb, and Rag [2], but upstream mTORC2 modulators remain largely elusive. Recently, regulation of mTORC2 activity by lysosome positioning [3] and mTORC2 complexes with other proteins [4] emerged as new potential mechanisms that coordinate mTORC2/Akt signaling.

The phosphatidylinositol 3 kinase (PI3K)/Akt pathway is essential in tumor initiation and progression [5]. Activated PI3K phosphorylates PI(4,5)P2 to form PI(3,4,5)P3, which induces plasma membrane recruitment of Akt through its N-terminal Pleckstrin homology domain (PH domain) [5, 6]. The membrane recruited Akt is phosphorylated in two crucial residues: threonine-308 (Thr-308) and serine-473 (Ser-473). PDK1 and mTORC2 are the main upstream kinases that phosphorylate Akt at Thr-308 and Ser-473, respectively [6]. Full activation of Akt requires both phosphorylation and mediates the phosphorylation of members of the forkhead box O (FOXO) family, glycogen synthase kinase 3 (GSK3), tuberous sclerosis complex 2 (TSC2), and many other substrates regulating a wide repertoire of signaling pathways [6]. Thus, the PI3K/Akt signaling activation and the Akt-substrate specificity are critical for tissue homeostasis. The hyperactivation of PI3K/Akt signaling or its deregulation leads to several pathological outcomes, such as neurological disorders, cancer, and drug resistance [5, 7–9]. Therefore, an active cancer research field investigates the underlying molecular mechanisms mediating Akt-activation and -substrate specificity.

NUAK1 (aka ARK5) is a Ser/Thr kinase member of the AMPK-related family, composed of 12 kinases related by sequence homology with the catalytic domain of the AMPK-α subunit [10]. Collectively, these kinases regulate cell adhesion, polarity, metabolism, and the response to different stresses, including energetic, osmotic, and oxidative stress [11]. The main post-translational modifications mediating NUAK1 activation are phosphorylation led by Liver Kinase B1 (LKB1) at the threonine 211 (Thr-211) [10] and by Akt at the serine 600 (Ser-600) [12]. Like Akt, NUAK1 promotes tumor initiation and progression through regulation of cell proliferation [13, 14], induction of cancer cell survival [12], cell migration [15], changes in cellular metabolism [16, 17], and oxidative stress regulation [18]. However, there is insufficient evidence for NUAK1 regulation and function under growth factor signaling or vice versa. Indeed, its regulation and targets remain still scarce.

Here, we report a new role of NUAK1 in cancer signaling. NUAK1 acts as a novel regulator of mTORC2 and its downstream target Akt. Our results indicated that NUAK1 coordinates mTORC2/Akt activity by controlling lysosome positioning and mTOR association with this organelle. In addition, NUAK1 is a novel kinase that directly phosphorylates Akt at Ser-473, inducing an early Akt-activation and -substrate specificity according to its subcellular location at early endosomes. Through these mechanisms, NUAK1 supports cancer cell survival; thus, inhibiting NUAK1, or combined inhibition with Akt or mTOR inhibitors, may be considered in cancer treatments.

## Results

### NUAK1 interacts with components of mTORC2

**Fig. 1:**
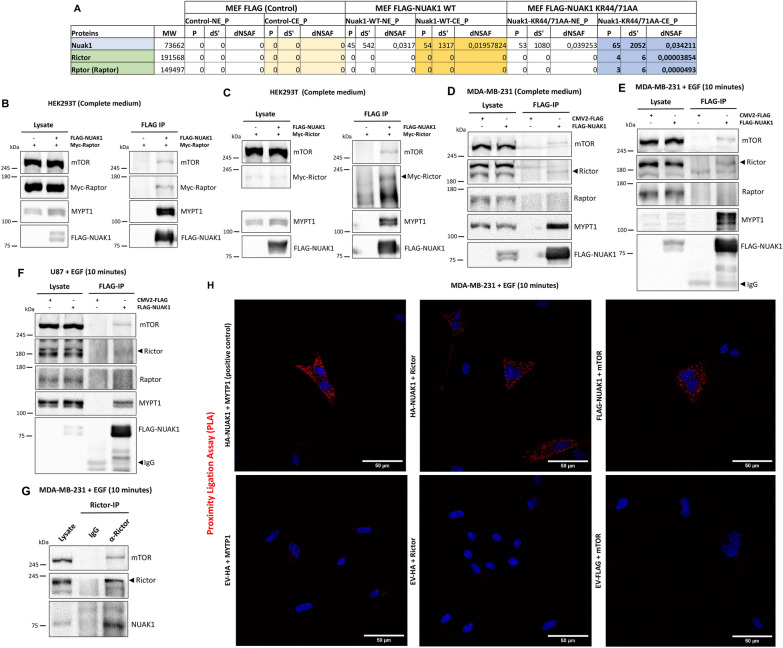
NUAK1 interacts with mTOR and Rictor but not with Raptor upon EGF stimulation. **A** Table shows total peptide count (P), the distributed spectra count (dS), and distributed normalized spectral abundance (dNSAF), observed for each identified protein in murine FLAG-NUAK1 WT and FLAG-NUAK1 KR44/71AA purifications (n = 3). NE, nuclear extract; CE, cytoplasmic extract. **B** Immunoblot (IB) of the Immunoprecipitation (IP) of human FLAG-NUAK1 WT and CoIP of endogenous mTOR, MYPT1 and exogenous Myc-Raptor in HEK293T cells. **C** IB of the IP of FLAG-NUAK1 WT and CoIP of endogenous mTOR, MYPT1 and exogenous Myc-Rictor in HEK293T cells. **D** IB of the IP of FLAG-NUAK1 WT and CoIP of endogenous mTOR, Rictor, Raptor and MYPT1 in MDA-MB-231 cells. **E**, **F** IB of the IP of FLAG-NUAK1 WT and CoIP of endogenous mTOR, Rictor, Raptor and MYPT1 from MDA-MB-231 (**E**) and U87 (**F**) cells serum-starved overnight before stimulation with EGF by 10 min. **G** IB of the IP of endogenous Rictor and CoIP of endogenous mTOR, and NUAK1 in MDA-MB-231 cells serum-starved overnight before stimulation with EGF by 10 min. **H** Proximity ligation assay (PLA) in MDA-MB-231 cells expressing HA-tagged NUAK1, FLAG-tagged NUAK1 or Empty vector (EV) (used as a negative control). Cells were serum-starved overnight and stimulated with EGF by 10 min (n = 3). Red dots indicate proximity of HA-NUAK1 with MYPT1 (Positive control), HA-NUAK1 with Rictor or FLAG-NUAK1 with mTOR. DAPI was used as a nuclear counterstain

Recently, new evidence suggested a critical role of NUAK1 in cancer cell signaling [19, 20]. We performed mass spectrometry analyses to identify molecular mechanisms associated with NUAK1 function. By Multidimensional Protein Identification Technology (MudPIT) of immunoprecipitated FLAG-Tagged murine NUAK1 WT and FLAG-Tagged murine NUAK1 KR44/71AA (cytoplasmic mutant) from immortalized Mouse Embryonic Fibroblast (iMEFs) [21], we identified Rictor and Raptor as novel potential cytoplasmic NUAK1 binding partners (Fig. 1A). The mass spectrometry data was validated by co-immunoprecipitation experiments in HEK293T cells, using the overexpression of exogenous FLAG-Tagged human NUAK1 WT and Myc-Tagged Raptor or Myc-Tagged Rictor (Fig. 1B and C). Nevertheless, we found that FLAG-Tagged NUAK1 interacts with endogenous mTOR and Rictor in MDA-MB-231 cells but not with Raptor (Fig. 1D). MYPT1, a known NUAK1 binding partner [22], was used as a positive control. Because both mTOR complexes coordinate growth factor signaling, we explored whether NUAK1 interacts with mTOR, Raptor, or Rictor after growth factor stimulation. FLAG-Tagged NUAK1 immunoprecipitation in MDA-MB-231 or U87 cells confirmed that NUAK1 interacts with mTOR and Rictor but not with Raptor upon EGF stimulation (Fig. 1E, F). Immunoprecipitation of endogenous Rictor and Proximity Ligation Assay (PLA) of NUAK1 with Rictor or mTOR confirmed the association between NUAK1 and mTORC2 (Fig. 1G, H). Altogether, our results suggested a functional association between NUAK1 and mTORC2.

### NUAK1 regulates mTOR accumulation at the lysosome and lysosome positioning

**Fig. 2:**
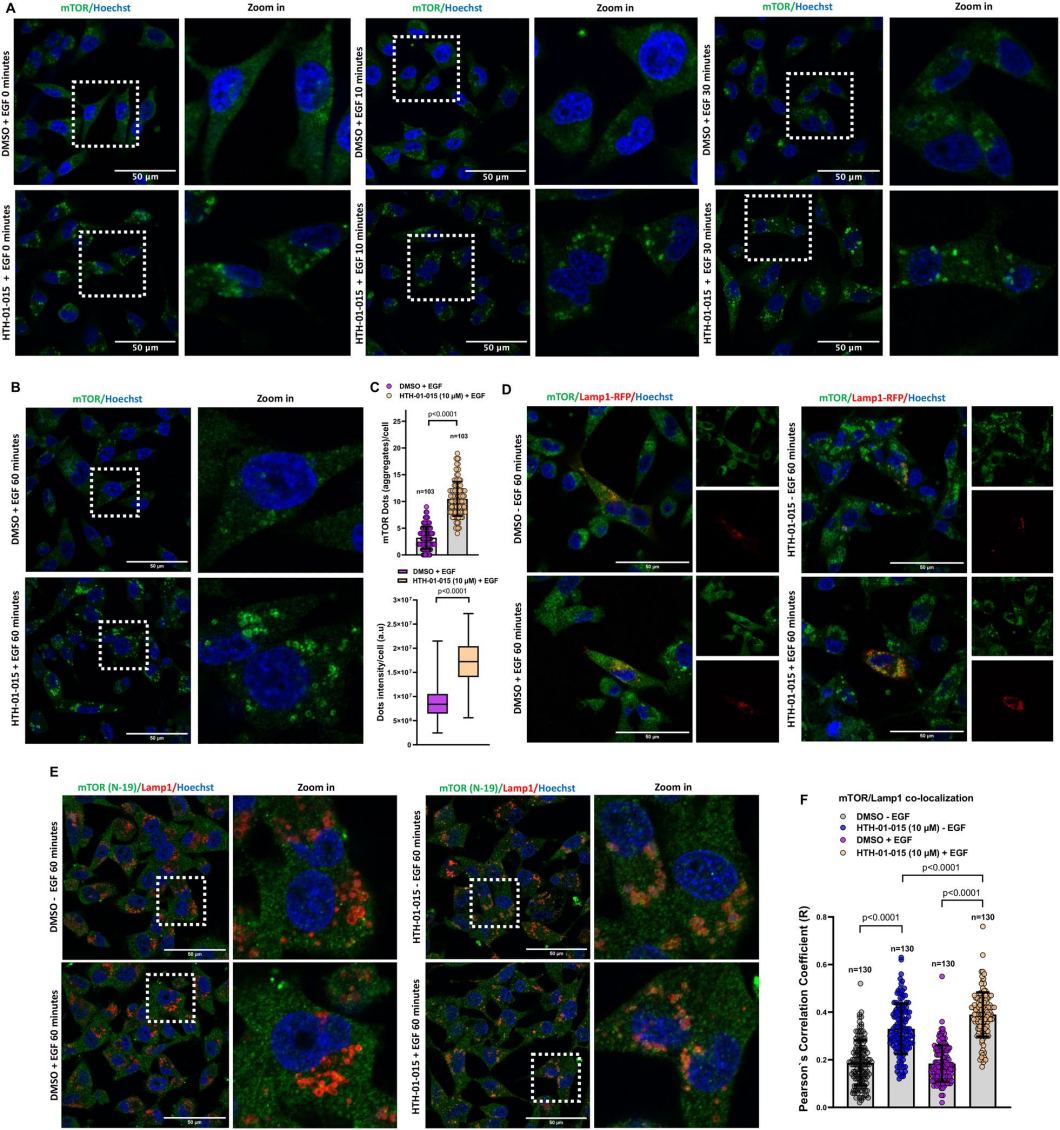
NUAK1 inhibition induces mTOR accumulation at the lysosome. **A** Representative confocal images of mTOR under NUAK1 inhibition. MDA-MB-231 cells were serum-starved overnight followed by 90 min of pretreatment with DMSO or HTH-01-015 (10 µM) before EGF stimulation for 0, 10, and 30 min. Green, mTOR; Blue, nuclei. **B** Representative confocal images of mTOR under NUAK1 inhibition. MDA-MB-231 cells were serum-starved overnight followed by 90 min of pretreatment with DMSO or HTH-01-015 (10 µM) and EGF-stimulated for 60 min. Green, mTOR; Blue, nuclei. **C** Quantification of the number of dots (upper) and intensity (lower) from **B**. Each bar represents the mean ± SD, Student t test. **D** Representative confocal images of mTOR and Lamp1-RFP after NUAK1 inhibition from non-stimulated or EGF-stimulated MDA-MB-231 cells for 60 min. Left, merge; Right, mTOR and Lamp1-RFP images. Green, mTOR; Red, Lamp1-RFP; Blue, nuclei. **E** Representative confocal images of endogenous mTOR (N-19) and Lamp1 in MDA-MB-231 cells after NUAK1 inhibition from non-stimulated or EGF-stimulated cells for 60 min. Left, merge; Right, zoom in. Green, mTOR (N-19); Red, Lamp1; Blue, nuclei. **F** Quantification of mTOR/Lamp1 co-localization from **E**. Each bar represents the mean ± SD, Student t test

**Fig. 3:**
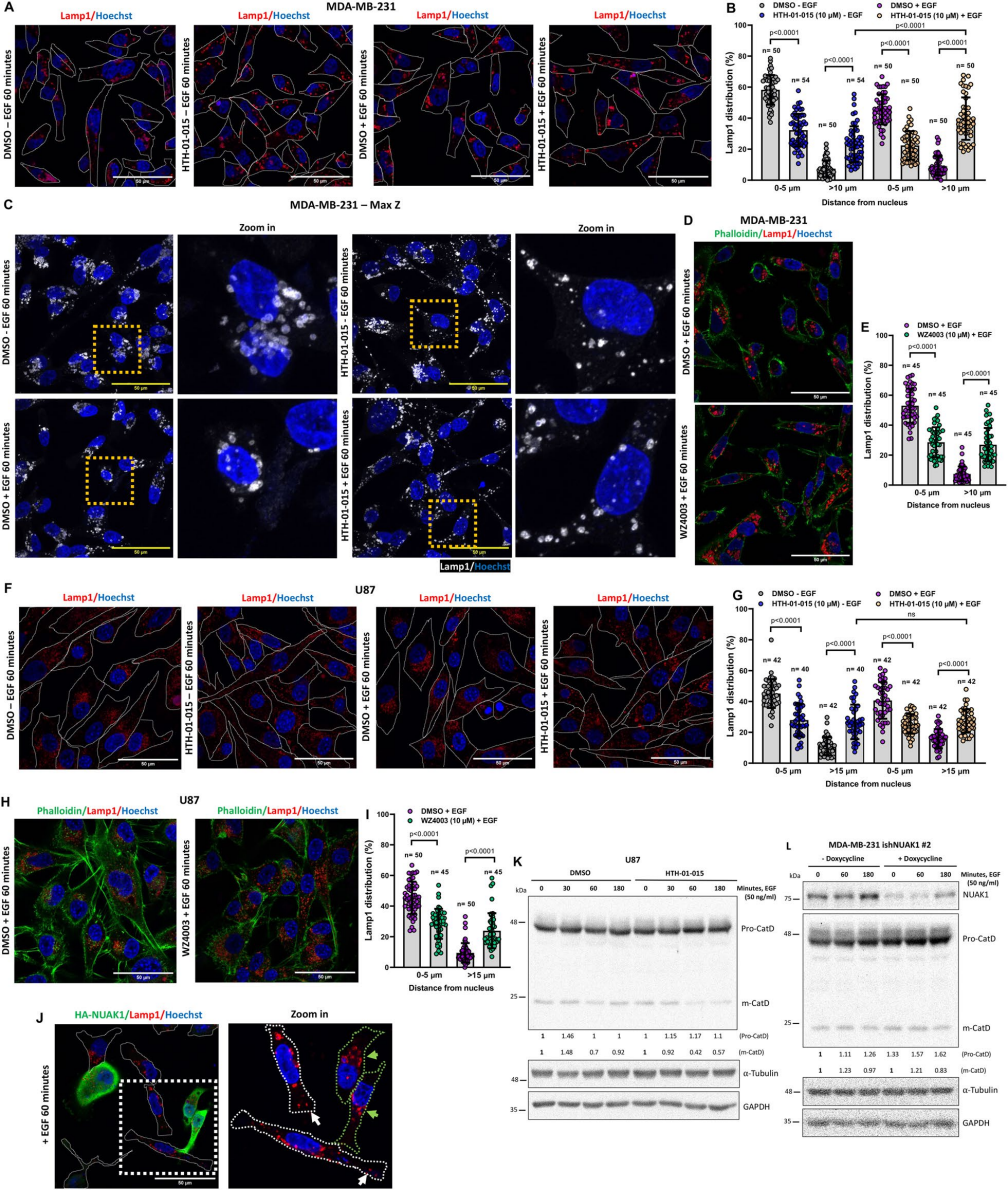
NUAK1 inhibition induces peripheral lysosomal positioning. **A**, **F** Representative confocal images of lysosomes using an anti-Lamp1 antibody (lysosome marker) in MDA-MB-231 (**A**) and U87 cells (**F**) under HTH-01-015 treatment. MDA-MB-231 and U87 cells were serum-starved overnight followed by 90 min of pretreatment with DMSO or HTH-01-015 (10 µM) and non-stimulated or EGF-stimulated for 60 min. Cells borders were marked with a boundary. Red, Lamp1; Blue, nuclei. **B**, **G** Quantification of the distribution of Lamp1^+^-lysosomes from **A** and **F**, respectively. Each bar represents the mean ± SD, Student t test. **C** Representative z-stack projections of Lamp1^+^-lysosomes in MDA-MB-231 cells. **D**, **H** Representative confocal images of lysosomes in MDA-MB-231 (**D**) and U87 cells (**H**) under WZ4003 treatment. Cells were serum-starved overnight followed by 90 min of pretreatment with DMSO or WZ4003 (10 µM) before stimulation with EGF for 60 min. Green, Phalloidin (F-actin); Red, Lamp1; Blue, nuclei. **E**, **I** Quantification of the distribution of Lamp1^+^-lysosomes from **D** and **H**, respectively. Each bar represents the mean ± SD, Student t test. **J** Representative confocal images of lysosomes in MDA-MB-231 expressing HA-tagged NUAK1 and stimulated with EGF for 60 min. Cells borders were marked with a boundary. Green, HA-NUAK1; Red, Lamp1; Blue, nuclei. **K** IB of Cathepsin D under NUAK1 inhibition in U87 cells. **L** IB of Cathepsin D under NUAK1 depletion in MDA-MB-231 cells

The role of mTORC2 signaling upon growth factor stimulation has been well-described, but upstream modulators and signaling crosstalk under these conditions still need to be better understood [23]. Thus, we investigated whether NUAK1 impacts the mTORC2 function by affecting the association between mTOR and Rictor. HTH-01-015, a specific well-validated NUAK1 inhibitor [22], did not affect mTOR/Rictor association after 5 or 60 min of EGF stimulation in MDA-MB-231 and U87 cells (Additional file 1: Fig. S1A–C). Recently, mTORC2 has been localized to different organelles, driving its activity and inducing Akt signaling activation [24]. In addition, lysosome biogenesis and positioning are critical regulators of its function and homeostasis [25]. Indeed, peripheral lysosomes induce a faster reactivation of mTORC2/Akt signaling upon growth factor stimulation [3]. Interestingly, the NUAK1 inhibitor induced a substantial accumulation of mTOR in MDA-MB-231 cells, significantly increasing the number and intensity of mTOR aggregates, opposite to the control cells showing homogeneous mTOR subcellular distribution (Fig. 2A–C). We then investigated whether the effect of NUAK1 inhibition was due to changes in mTOR association with the lysosomes. Using different endogenous mTOR antibodies and overexpression of Lamp1-RFP or an antibody against endogenous Lamp1 (lysosomal marker), we found that NUAK1 inhibition (Fig. 2D–F) and NUAK1-silencing by inducible NUAK1 small hairpin RNA (ishNUAK1, Additional file 1: Fig. S2A) induce mTOR accumulation at the lysosomes that increases after EGF stimulation. In addition, NUAK1 inhibition reduced mTOR/Rab5+ early-endosome association (Additional file 1: Fig. S2B, C). Interestingly, HTH-01-015 or WZ4003 (NUAK1/2 inhibitor) also affected the subcellular distribution of the lysosomes, inducing peripheral lysosomal positioning in MDA-MB-231 (Fig. 3A–E) and U87 cells (Fig. 3F–I), but did not affect the subcellular distribution of Rab5+ early-endosomes (Additional file 1: Fig. S3). In MDA-MB-231 cells, NUAK1 affected lysosomal positioning in non-stimulated or EGF-stimulated cells (Fig. 3A–E). Like the mTOR accumulation shown in Fig. 2E, F, the EGF stimulation also increased the peripheral lysosomal positioning (Fig. 3A, B). In U87 cells, NUAK1 inhibition was enough to promote changes in lysosomal distribution, inducing their peripheral location (Fig. 3F–I). In contrast, after EGF stimulation, NUAK1 overexpression maintained the lysosome at the perinuclear region (Fig. 3J). To confirm the effect of NUAK1 on the lysosomal positioning, we analyzed cathepsin D maturation (m-CatD). The perinuclear lysosomes are more acidic than the peripheral ones [26]. Thus, the maturation of Cathepsin D is higher in more acidic perinuclear lysosomes [27]. NUAK1 inhibition with HTH-01-015 caused a reduction of Cathepsin D maturation (m-CTSD) in U87 cells (Fig. 3K), and ishNUAK1 induced the accumulation of pro-Cathepsin D (pro-CatD) in MDA-MB-231 cells (Fig. 3L), confirming the role of NUAK1 in inducing peripheral lysosome distribution. Therefore, NUAK1 regulates the functional lysosome positioning and the mTOR subcellular distribution, suggesting a fine-tuned regulation of mTOR function.

### NUAK1 induces early activation of Akt

**Fig. 4:**
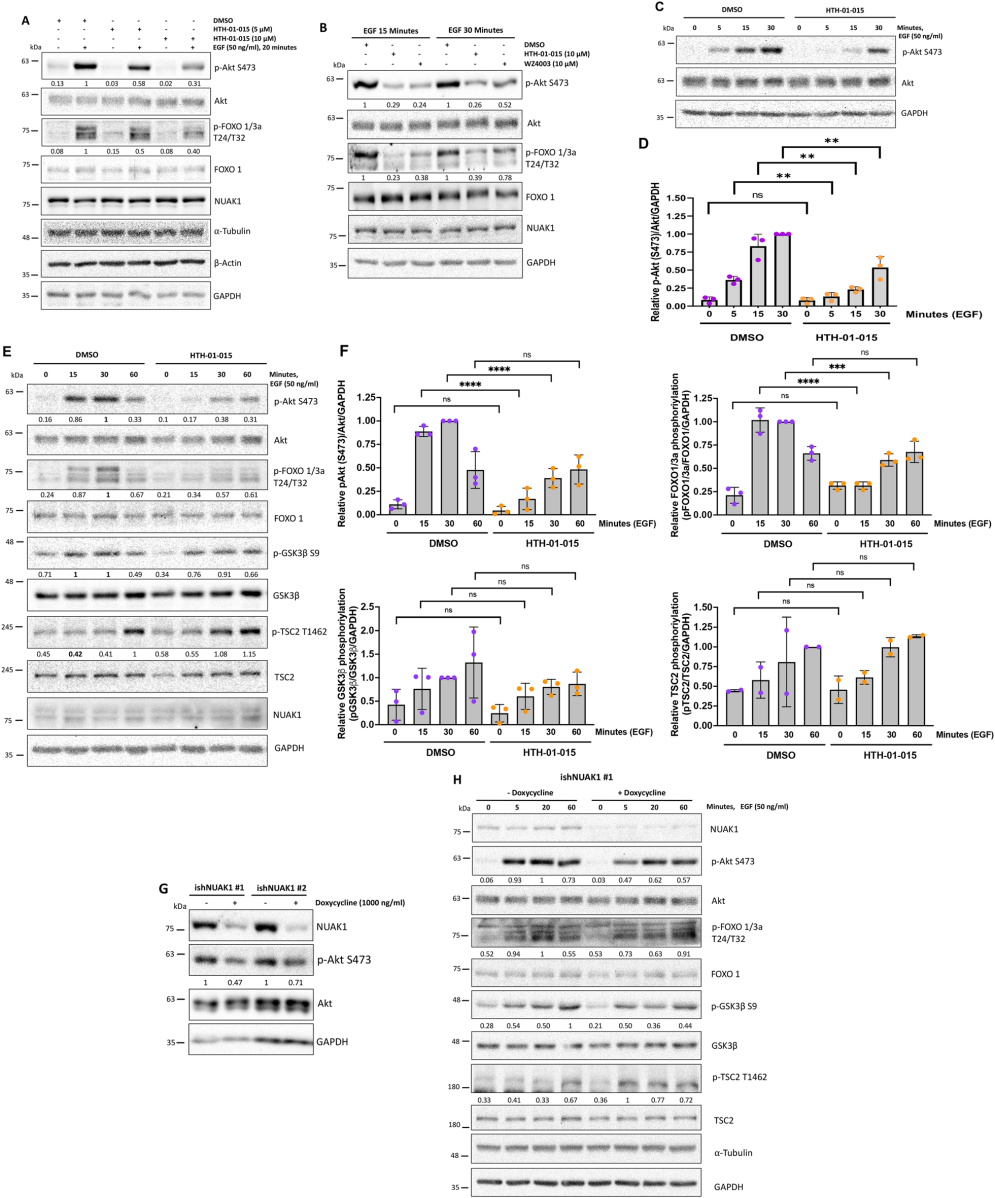
NUAK1 regulates Akt signaling under growth factors stimulation. **A** IB of Akt signaling under NUAK1 inhibition in MDA-MB-231 cells serum-starved overnight followed by 1-h of pretreatment with DMSO or HTH-01-015 (5 µM and 10 µM) before stimulation with EGF for 20 min. **B** IB of Akt signaling under NUAK1/2 inhibitors in MDA-MB-231 cells serum-starved overnight followed by 1-h of pretreatment with DMSO, HTH-01-015 (10 µM) or WZ4003 (10 µM) before stimulation with EGF for 15 and 30 min. **C** IB of Akt signaling under NUAK1 inhibition in MDA-MB-231 cells serum-starved overnight followed by 1-h of pretreatment with DMSO or HTH-01-015 (10 µM) before stimulation with EGF for 0, 5, 15, and 30 min. **D** Quantification of Akt phosphorylation at Ser-473 from **C**. Each bar represents the mean ± SD, n = 3. Data from 3 independent were analyzed by one-way ANOVA followed by Turkey’s multiple comparison test. **E** IB of Akt signaling under NUAK1 inhibition in MDA-MB-231 cells serum-starved overnight followed by 1-h of pretreatment with DMSO or HTH-01-015 (10 µM) before stimulation with EGF for 0, 15, 30 and 60 min. **F** Quantification of Akt phosphorylation at Ser-473 from **E**. Each bar represents the mean ± SD, n = 3. Data from 3 independent experiments (two for pTSC2) were analyzed by one-way ANOVA followed by Turkey’s multiple comparison test. **G** IB of Akt Ser-473 phosphorylation using inducible shRNAs for NUAK1. MDA-MB-231 cells stable expressing inducible shRNA vectors for NUAK1 [#1 (left) and #2 (right)] were pretreated with doxycycline or vehicle (used as a negative control) by 4 days. **H** IB of Akt signaling using inducible shRNA for NUAK1. MDA-MB-231 cells stable expressing inducible shRNA vector for NUAK1 #1 were pretreated with doxycycline or vehicle (used as a negative control) for 3 days followed by serum starvation overnight (with or without doxycycline) before stimulation with EGF. α-tubulin and/or GAPDH were used as loading controls

According to the association of NUAK1 with mTORC2 and NUAK1’s effect on lysosome positioning, we expected that NUAK1 affects the mTORC2 signaling. mTORC2 is the main upstream kinase for the Akt Ser-473 phosphorylation, used as a marker of mTORC2 activity [6]. Therefore, we investigated whether NUAK1 is involved in the Akt signaling activation. We found that HTH-01-015 dose-dependently inhibited Akt activation, evidenced by a decrease in the Akt Ser-473 phosphorylation in EGF-stimulated MDA-MB-231 cells (Fig. 4A). WZ4003, a dual NUAK1/2 inhibitor, showed a similar result (Fig. 4B). We next analyzed the effect of NUAK1 on the phosphorylation of Akt and its main downstream targets at different time points of the EGF stimulation. Interestingly, we observed that NUAK1 inhibition affects the early activation of Akt (Fig. 4C, D), starting to reactivate after 30 min. The effect of NUAK1 inhibition on the Akt activation correlated with a substantial reduction in the phosphorylation of FOXO1/3a (Thr-24/Thr-32) but not the phosphorylation of TSC2 (Thr-1462) (Fig. 4E, F). Surprisingly, NUAK1 slightly affected GSK3β Ser-9 phosphorylation under growth factor stimulation (Fig. 4E, F). However, NUAK1 inhibition reduced GSK3β Ser-9 phosphorylation under normal growth conditions and oxidative stress (Additional file 1: Fig. S4A, B). We also observed the early effect of NUAK1 on the Akt Ser-473 phosphorylation in the U87 and SW480 cancer cell lines (Additional file 1: Fig. S4C, D). To further support the role of NUAK1 in the Akt signaling, we used two different ishNUAK1. NUAK1 depletion also reduced the phosphorylation of Akt, although the ishNUAK1#1 has a stronger effect than the ishNUAK1 #2 (Fig. 4G). Consistently, ishNUAK1 #1 reduced the phosphorylation of Akt at Ser-473 and its main downstream targets upon growth factor stimulation (Fig. 4H).

Additionally, we investigated whether the NUAK1 effect on the Akt signaling is conserved under different cellular conditions. We found that NUAK1 inhibition decreased the Akt Ser-473 phosphorylation and the Akt signaling under insulin stimulation (Additional file 1: Fig. S4E), normal growth (Fig. 4G and Additional file 1: Fig. S4A), and oxidative stress conditions (Additional file 1: Fig. S4B), suggesting that NUAK1’s role in Akt signaling is conserved across different cellular contexts. Altogether, our data identified that NUAK1 is involved in the early and maximal activation of the Akt signaling, resulting in the phosphorylation of FOXO1/3a (Thr-24/Thr-32) but not TSC2 phosphorylation.

### Signaling crosstalk between NUAK1 and mTORC2 regulates the EGF-dependent activation of Akt

**Fig. 5:**
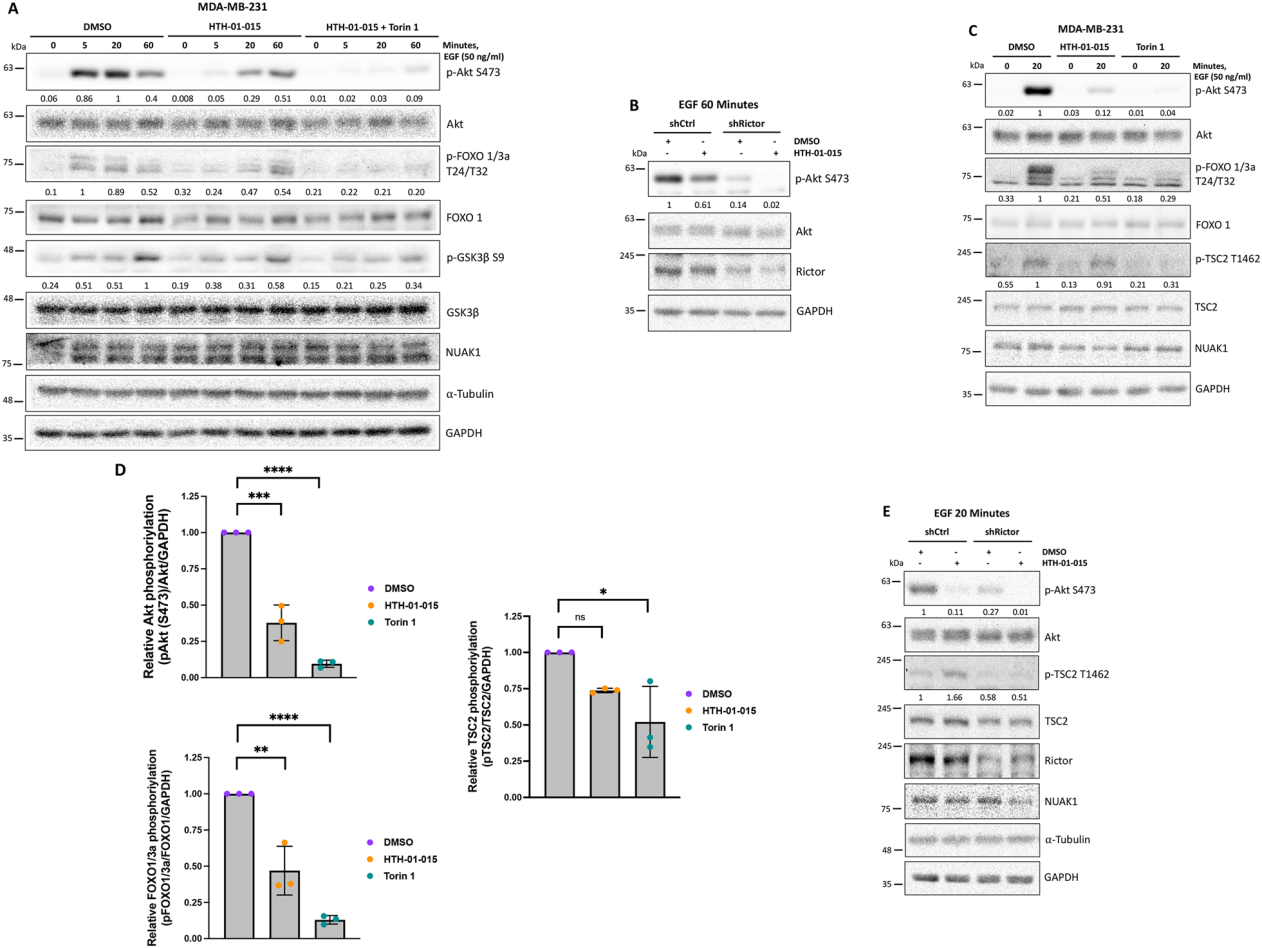
Comparative of NUAK1 inhibition versus mTOR inhibition on Akt signaling. **A** IB of combined inhibition of NUAK1 and mTOR on Akt signaling. MDA-MB-231 cells were serum-starved overnight followed by 1-h of pretreatment with DMSO, HTH-01-015 (10 µM) or HTH-01-015 (10 µM) plus Torin1 (100 nM) before stimulation with EGF. **B** IB of Akt Ser-473 phosphorylation under NUAK1 inhibition in MDA-MB-231 shCtrl and MDA-MB-231 shRictor cells. Stable cells were serum-starved overnight followed by 1-h of pretreatment with DMSO or HTH-01-015 (10 µM) before stimulation with EGF for 60 min. **C** IB of NUAK1 and mTOR effect on Akt signaling. MDA-MB-231 cells were serum-starved overnight followed by 1-h of pretreatment with DMSO, HTH-01-015 (10 µM) or Torin1 (100 nM) before stimulation with EGF for 0 and 20 min. **D** Quantification of Akt signaling pathway from **C**. Each bar represents the mean ± SD, n = 3. Data from 3 independent experiments at 20 min of EGF stimulation were analyzed by student t test. **E** IB of Akt/TSC2 signaling under NUAK1 inhibition in MDA-MB-231 shCtrl and MDA-MB-231 shRictor cells. Stable cells were serum-starved overnight followed by 1-h pretreatment with DMSO or HTH-01-015 (10 µM) before stimulation with EGF for 20 min. GAPDH and/or α-tubulin were used as loading controls

To understand the role and relevance of NUAK1 in the mTORC2/Akt signaling, we compared the effect of NUAK1 inhibition versus mTOR inhibition on Akt signaling. We first explored whether mTORC2 is responsible for the late activation of Akt induced under NUAK1 inhibition (60 min after EGF stimulation). By combining treatment with HTH-01-015 and Torin 1 or a specific shRNA against Rictor (shRictor), we confirmed that mTORC2 activity is responsible for the late activation of Akt (Fig. 5A, B). However, both kinases were strongly required for the Akt phosphorylation and activation after 20 min of EGF stimulation (Fig. 5C, D). We confirmed that HTH-01-015-dependent inhibition of Akt only significantly affected FOXO1/3a phosphorylation (Fig. 5C, D). However, Torin 1-dependent inhibition of Akt reduced the phosphorylation of both FOXO1/3a and TSC2 (Fig. 5C, D). To confirm the difference between NUAK1 and mTORC2 on their regulation of the Akt downstream targets, we inhibited mTORC2 by using shRictor. Rictor depletion reduced the Akt Ser-473 phosphorylation induced by 20 min of stimulation with EGF, which correlated with a reduction in TSC2 phosphorylation (lane 1 vs. lane 3, Fig. 5E). Instead, like in the ishNUAK1 experiments (see Fig. 4H), the inhibition of Akt Ser-473 phosphorylation by pharmacological inhibition of NUAK1 correlated with increased TSC2 phosphorylation that Rictor depletion blocked (lane 2 vs. lane 4, Fig. 5E). Thus, NUAK1’s effect on Akt signaling may be compartmentalized, explaining its failure to affect TSC2 phosphorylation. In addition, because NUAK1 inhibition resulted in a mTORC2-dependent late activation of Akt, our results suggest a fine-tuned regulation of the Akt activity through signaling crosstalk between NUAK1 and mTORC2.

### NUAK1 resides at the early endosomes

**Fig. 6:**
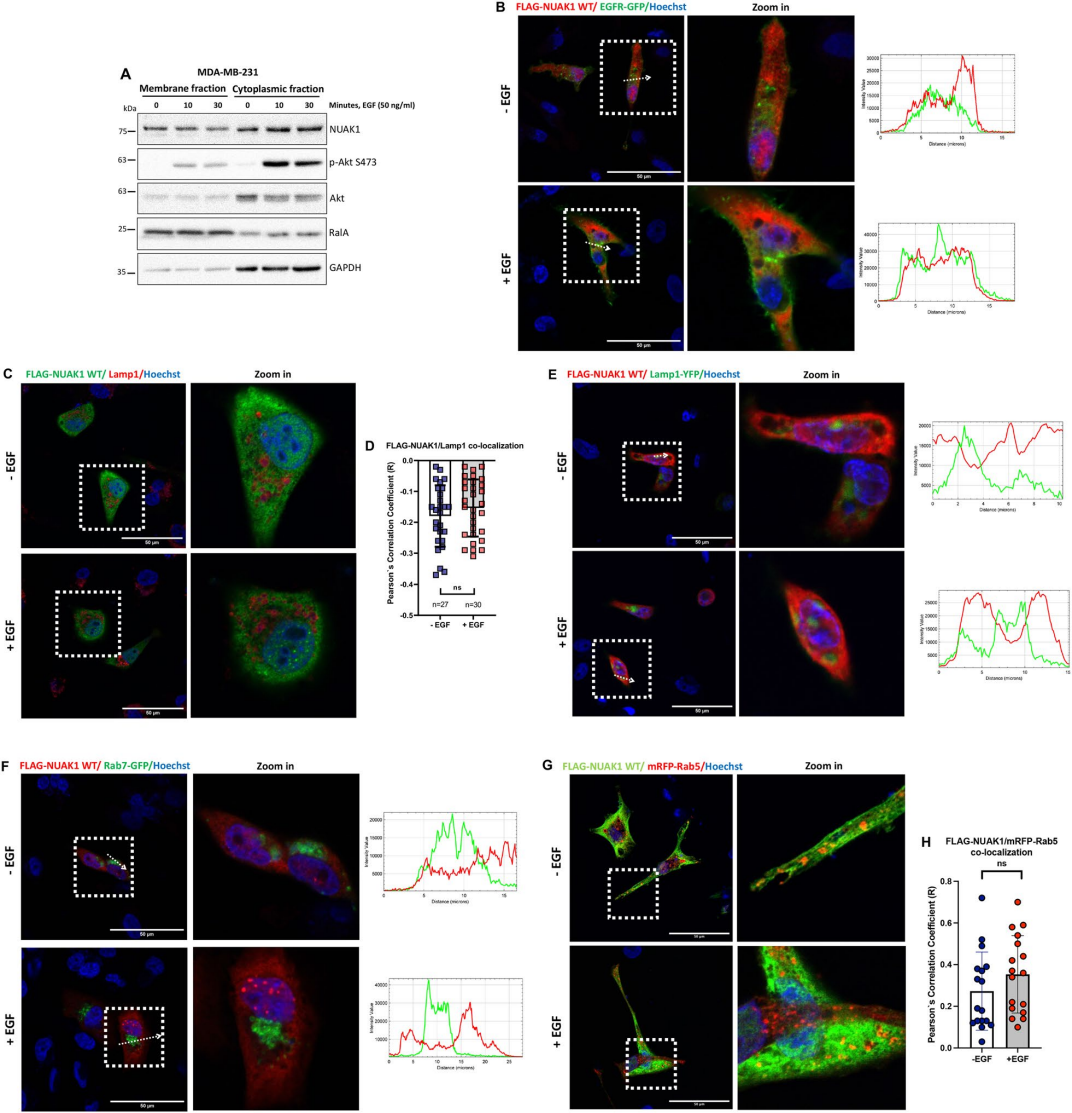
NUAK1 is localized at early-endosomes but not at the PM, lysosomes, and late-endosomes. **A** IB of NUAK1 subcellular location. Subcellular fractionation of the membrane and cytoplasmic fractions of MDA-MB-231 cells after 0, 10 and 30 min of EGF stimulation. p-Akt S473 was used as a positive control. RalA was used as a control for the membrane fraction. GAPDH was used as a control for the cytoplasmic fraction. **B**, **C** Immunofluorescence (IF) of MDA-MB-231 cells after 0 (−EGF) and 15 (+EGF) minutes of EGF stimulation. **B** Left, Representative confocal images for FLAG-NUAK1 WT and EGFR-GFP, arrows indicate the distance (µm) analyzed; Right, Histogram of the fluorescence intensity profile across the arrow for both red and green channels. Red, FLAG-Tagged-NUAK1; Green, EGFR-GFP (PM marker); Blue, nuclei. **C** Representative confocal images for FLAG-NUAK1 WT and Lamp1. Green, FLAG-Tagged-NUAK1; Red, endogenous Lamp1 (lysosome marker); Blue, nuclei. **D** Quantification of FLAG-NUAK1/Lamp1 co-localization from **C**. Each bar represents the mean ± SD, not significant (ns), Student t test. **E** IF of MDA-MB-231 cells after 0 (−EGF) and 30 (+EGF) minutes of EGF stimulation. Left, Representative confocal images for FLAG-NUAK1 WT and Lamp1-YFP, arrows indicate the distance (µm) analyzed; Right, Histogram of the fluorescence intensity profile across the arrow for both red and green channels. Red, FLAG-Tagged-NUAK1; Green, Lamp1-YFP (lysosome marker); Blue, nuclei. **F** IF of MDA-MB-231 cells after 0 (−EGF) and 15 (+EGF) minutes of EGF stimulation. Left, Representative confocal images for FLAG-NUAK1 WT and Rab7-GFP, arrows indicate the distance (µm) analyzed; Right, Histogram of the fluorescence intensity profile across the arrow for both red and green channels. Red, FLAG-Tagged-NUAK1; Green, Rab7-GFP (late-endosome marker); Blue, nuclei. **G** IF of MDA-MB-231 cells after 0 (−EGF) and 30 (+EGF) minutes of EGF stimulation. Representative confocal images for FLAG-NUAK1 WT and mRFP-Rab5. Green, FLAG-Tagged-NUAK1; Red, mRFP-Rab5 (early-endosome marker); Blue, nuclei. **H** Quantification of FLAG-NUAK1/mRFP-Rab5 co-localization from **G**. Each bar represents the mean ± SD, not significant (ns), Student t test

Restrictive Akt activity at the plasma membrane (PM) or endomembranes provides a model for Akt-activation and -substrate specificity [28]. Because NUAK1-dependent regulation of Akt signaling did not affect TSC2 phosphorylation, we explored whether NUAK1 subcellular localization determines how it regulates Akt activation. Previously, we found that NUAK1 is localized in the nucleus and cytoplasm [21]. However, NUAK1’s association with membranes was not reported. By using cell fractionation, we found that NUAK1 is in the membrane and cytoplasmic fractions independently of EGF stimulation, suggesting a pool of NUAK1 at the PM or endomembranes (Fig. 6A). To validate NUAK1’s association with membranes, we analyzed NUAK1 co-localization with PM or endomembrane markers by confocal microscopy. First, we found that NUAK1 does not co-localize with EGFR, a common PM marker (Fig. 6B), suggesting that NUAK1 regulates Akt signaling at endomembranous system. Notably, NUAK1 is excluded from Lamp1+-labeled lysosomes from non-stimulated or EGF-stimulated MDA-MB-231 cells (Fig. 6C-E). The lysosome is the main organelle where mTORC1 signaling is regulated by many signals (amino acids and growth factors), including the Akt-dependent phosphorylation and inhibition of TSC2 [29]. Therefore, NUAK1 absence at the lysosome, mTOR accumulation at the lysosome, and lysosome peripheral positioning upon NUAK1 inhibition may explain why NUAK1 is not involved in the Akt-dependent phosphorylation of TSC2. The early endosome has been recognized as a new compartment for Akt activation, regulating GSK3β and FOXO1/3a phosphorylation under growth factors [30, 31]. Like the lysosome exclusion, NUAK1 is excluded from the Rab7+-labeled late-endosomes (Fig. 6F) but co-localized with the exogenous and endogenous Rab5+-labeled early-endosomes either during serum starvation or EGF stimulation (Fig. 6G, H and Additional file 1: Fig. S5). Altogether, our results suggest that NUAK1 subcellular localization and its regulation of mTOR association with the lysosome determines Akt-activation and -substrate specificity. Thus, NUAK1 at the early endosome may regulate Akt specificity for FOXO1/3a. On the other hand, NUAK1-dependent mTOR association with the lysosome may account for the late activation of Akt and the phosphorylation of TSC2.

### NUAK1 is a novel kinase that directly phosphorylates Akt at Ser-473

**Fig. 7:**
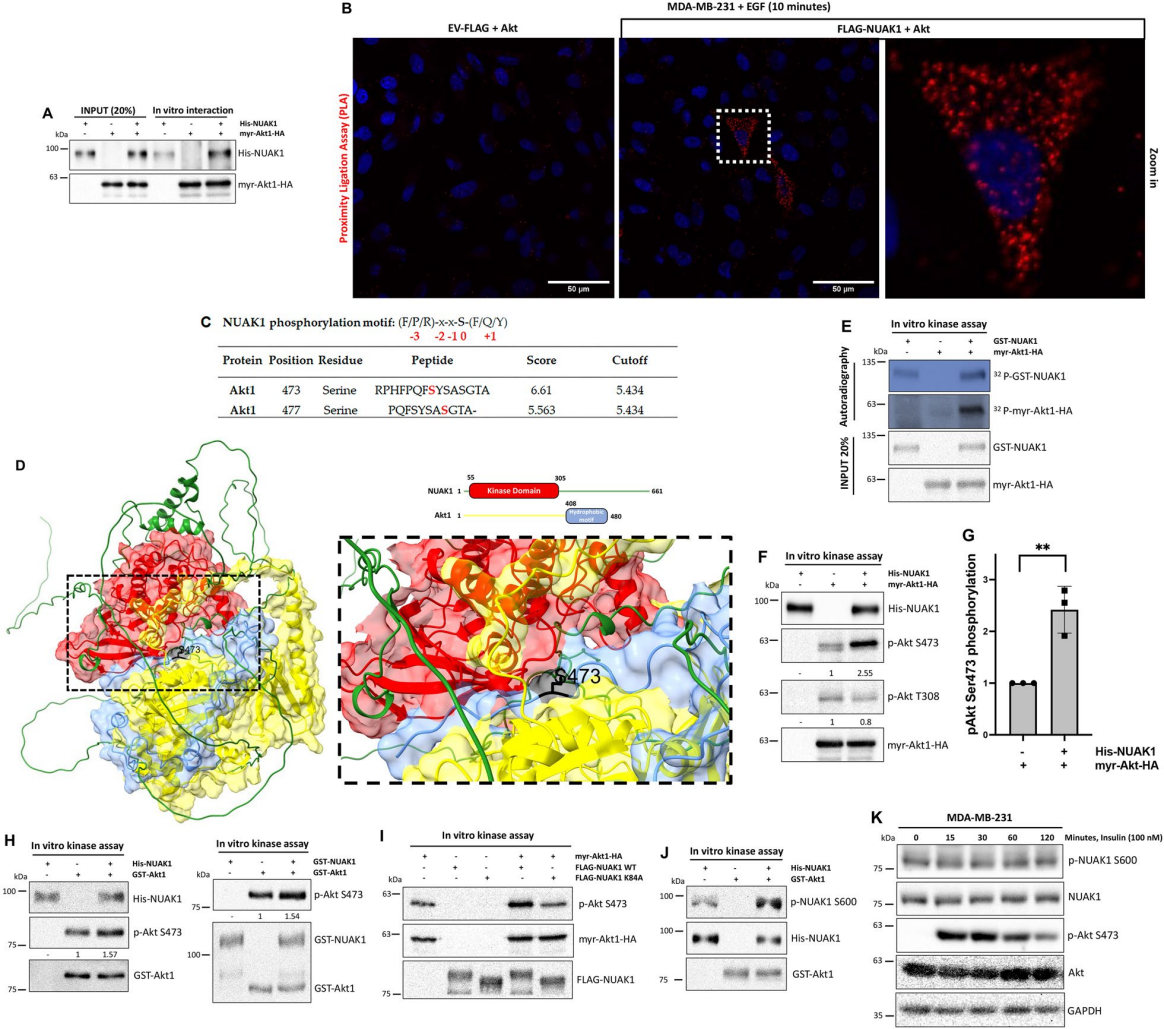
NUAK1 interacts with Akt and phosphorylates it at Ser-473. **A** IB of NUAK1 and Akt1 in vitro interaction. Recombinant His-tagged NUAK1 and immunoprecipitated HA-tagged Akt1 were used. **B** Proximity ligation assay (PLA) in MDA-MB-231 cells expressing FLAG-tagged NUAK1 or Empty vector (EV) (used as a negative control) were serum-starved overnight and stimulated with EGF by 10 min (n = 3). Red dots indicate proximity of FLAG-NUAK1 and Akt. DAPI was used as a nuclear counterstain. **C** Table of putative phosphoresidues phosphorylated by NUAK1 on Akt1. A consensus phosphorylation motif for NUAK1 obtained from GPS5.0 was used. **D** Molecular docking between NUAK1-Akt1. NUAK1 kinase domain (Red), Akt hydrophobic motif (Blue), and Ser-473 (Black) are represented. **E** Autoradiography of in vitro kinase assay using recombinant active GST-tagged NUAK1 and purified Akt1 HA-tagged. **F** IB of in vitro kinase assay using recombinant active His-tagged NUAK1 and purified Akt1 HA-tagged. Akt Ser-473 and Thr-308 phosphorylation were determined using specific antibodies. **G** Quantification of Akt S473 phosphorylation from **F**. Each bar represents the mean ± SD, n = 3. Data from 3 independent experiments were analyzed by Student t test. **H** IB of in vitro kinase assays using recombinant His-tagged NUAK1 and recombinant GST-tagged Akt1 (left) or recombinant active GST-tagged NUAK1 and recombinant GST-tagged Akt1 (right), respectively. **I** IB of in vitro kinase assays using purified FLAG-NUAK1 WT or FLAG-NUAK1 K84A (kinase-dead) and purified Akt1 HA-tagged. **J** IB of in vitro kinase assay using recombinant active GST-tagged Akt1 and recombinant His-tagged NUAK1. NUAK1 Ser-600 phosphorylation was determined using a specific antibody. All kinase assays were performed at least three times, except the kinase assay in **J**, that was performed two times. **K** IB of Akt signaling and NUAK1 phosphorylation at Ser-600 after insulin stimulation. GAPDH was used as loading control

The above results could explain the compartmentalized effect of NUAK1 on the Akt downstream substrates. However, we cannot discard that the effect of NUAK1 inhibition on the mTOR subcellular localization is responsible for the early reduction of the growth factor-dependent activation of Akt. In addition to mTORC2, new upstream kinases responsible for the phosphorylation of Akt at Ser-473 have been reported [32–34]. However, the cellular context and how those mechanisms are integrated with the mTORC2 signaling need to be better understood. Early studies showed that Akt interacts with NUAK1 and directly phosphorylates it at Ser-600 under glucose starvation [12]. We first confirmed that NUAK1 interacts with Akt in vitro (Fig. 7A). By a Proximity Ligation Assay (PLA), we validated the interaction between these proteins during EGF stimulation in MDA-MB-231 cells (Fig. 7B). Bioinformatical predictions suggested that NUAK1 phosphorylates Akt at Ser-473 and Ser-477 (Fig. 7C). Based on the relevance of the Akt phosphorylation at Ser-473, we initially performed molecular docking to explore whether the interaction between NUAK1 and Akt makes possible the phosphorylation of this residue. We used NUAK1 (O60285) and Akt1 (P31749) 3D structures from the Alpha Fold Protein Structure Database. Interestingly, the NUAK1 kinase domain (Red) directly interacts with the hydrophobic motif (HM) of Akt1 (blue), including the Ser-473 (Black) (Fig. 7D). Therefore, we evaluated whether NUAK1 phosphorylates Akt by a radioactive in vitro kinase assay. We used recombinant human GST-NUAK1 and immunoprecipitated Akt1-HA from cell lysates, suggesting that NUAK1 phosphorylated Akt1 and vice versa (Fig. 7E). Because the radioactive experiment could not discard that NUAK1 is inducing Akt autophosphorylation, we performed a non-radioactive in vitro kinase assay using recombinant human His-NUAK1, immunoprecipitated Akt1-HA and antibodies against phospho-Ser-473 and phospho-Thr-308 Akt, demonstrating that NUAK1 specifically phosphorylates the Ser-473, but not the Thr-308 of Akt1 (Fig. 7F, G). In vitro kinase assays using recombinant NUAK1 and Akt1 proteins (Fig. 7H) and a kinase-dead (KD) mutant of human NUAK1 (hNUAK1 K84A) (Fig. 7I and Additional file 1: Fig. S6) confirmed that NUAK1 directly phosphorylates Akt at Ser-473. Additionally, we validated that Akt1 phosphorylates NUAK1 at Ser-600 in vitro (Fig. 7J). However, growth factor-dependent stimulation of Akt does not induce the phosphorylation of NUAK1 at Ser-600 (Fig. 7K), suggesting that the phosphorylation of NUAK1 by Akt or vice versa depends on the cellular context.

### NUAK1/Akt/FOXO1/3a axis regulates the expression of p21CIP1, p27KIP1, FoxM1, and cancer cell survival upon growth factor stimulation

**Fig. 8:**
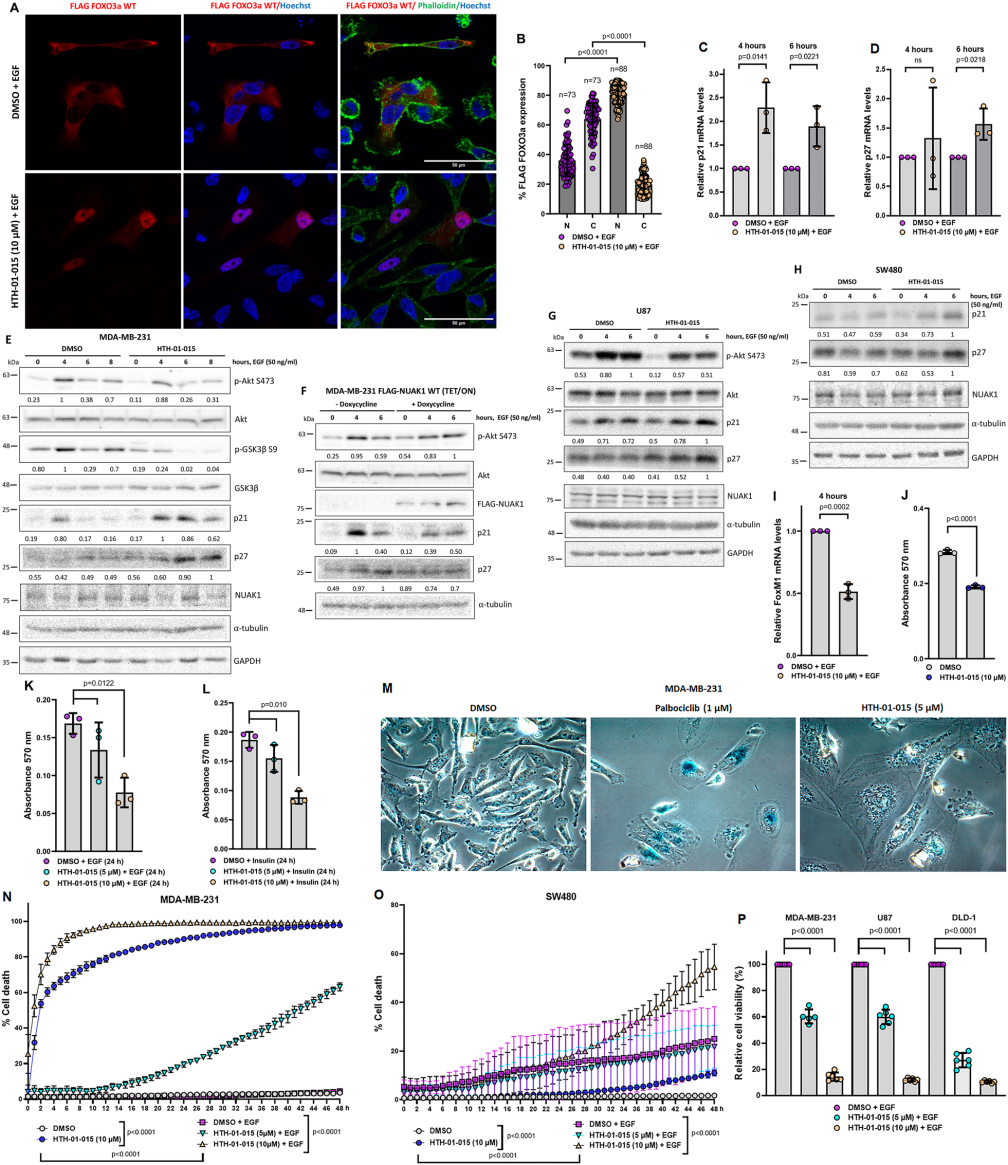
NUAK1 regulates FOXO3a subcellular localization, the expression of p21^CIP1^, p27^KIP1^, FoxM1, and cancer cell survival. **A** Representative confocal images of FLAG-tagged FOXO3a under NUAK1 inhibition. MDA-MB-231 cells expressing FLAG-tagged FOXO3a were serum-starved overnight followed by 1-h of pretreatment with DMSO or HTH-01-015 (10 µM) before 60 min of stimulation with EGF. Red, FLAG FOXO3a; Green, Phalloidin (F-actin); Blue, nuclei. **B** Quantification in % of FLAG-tagged FOXO3a expression from MDA-MB-231 IF experiments from **A**. N = nuclear fraction. C = Cytoplasmic fraction. Each bar represents the mean ± SD, Student t test. **C**–**E** p21 and p27 expression under NUAK1 inhibition. MDA-MB-231 cells were serum-starved overnight followed by 1-h of pretreatment with DMSO or HTH-01-015 (10 µM) before stimulation with EGF for 4 and 6 h for mRNA levels (**C**, **D**) (each bar represents the mean ± SD. Student t test, n = 3), and 0, 4, 6 and 8 h for protein levels (**E**). **F** IB of p21 and p27 expression under NUAK1 overexpression. MDA-MB-231 cells stable for FLAG-tagged NUAK1 inducible expression were pretreated with doxycycline or vehicle (used as a negative control) by 12 h followed by serum-starved overnight (with or without doxycycline) before stimulation with EGF for 0, 4 and 6 h. **G**, **H** IB of p21 and p27 expression under NUAK1 inhibition in U87 (**G**) and SW480 (**H**) cells serum-starved overnight followed by 1-h of pretreatment with DMSO or HTH-01-015 (10 µM) before stimulation with EGF for 0, 4 and 6 h. All IB are representative of at least three independent experiments. GAPDH and/or α-tubulin were used as loading controls. **I** FoxM1 mRNA levels under NUAK1 inhibition. MDA-MB-231 cells were serum-starved overnight followed by 1-h of pretreatment with DMSO or HTH-01-015 (10 µM) before stimulation with EGF for 4 h. Each bar represents the mean ± SD, Student t test, n = 3. **J** Quantification of the crystal violet staining to evaluate NUAK1 effect on cell number under complete medium. MDA-MB-231 cells were treated with DMSO or HTH-01-015 (10 µM) for 24 h. Each bar represents the mean ± SD, Student t test, n = 3. **K**, **L** Quantification of the crystal violet staining to evaluate NUAK1 effect on cell number under EGF- or insulin-stimulation. MDA-MB-231 cells were serum-starved overnight followed by 1-h pretreatment with DMSO or HTH-01-015 (5 µM or 10 µM) before stimulation with EGF (**K**) or Insulin (**L**) for 24 h. Each bar represents the mean ± SD, one-way ANOVA, n = 3. **M** NUAK1 effect on senescence. MDA-MB-231 cells were treated with DMSO, Palbociclib (1 µM) (used as a positive control) or HTH-01-015 (5 µM) for 4 days (n = 3). **N**, **O** NUAK1 effect on cell death in MDA-MB-231 (**N**) and SW480 (**O**) cells under normal growth conditions or EGF stimulation using Incucyte. Each bar represents the mean ± SD, Student t test, n = 5 or one‐way ANOVA, n = 5. **P** NUAK1 effect on cell viability in spheroids from MDA-MB-231, U87, and DLD-1 cells. Spheroids were pretreated with DMSO or HTH-01-015 (5 µM or 10 µM) before stimulation with EGF for 96 h. Each bar represents the mean ± SD, one‐way ANOVA, n = 6

To further investigate the functional relevance of the NUAK1/Akt signaling, we focused on the FOXO pathway. The FOXO transcription factors regulate the expression of essential genes for cell proliferation, cell death, senescence, angiogenesis, cell migration, and metastasis [35]. Due to their functions and regulations, FOXO family members are tumor suppressors. Oncogenic signals, such as the Akt pathway, phosphorylate FOXO1 (Thr-24, Ser-256, and Ser-319), FOXO3a (Thr-32, Ser-253, and Ser-315), FOXO4 (Thr28, Ser-193, and Ser-258) and FOXO 6 (Thr-26, and Ser-184). The phosphorylation of FOXO1 and FOXO3a induces FOXO1/3a cytoplasmic sequestration, abolishing their transcriptional activity [35]. Thus, we explored NUAK1’s effect on FOXO3a subcellular localization and the expression of p21CIP1 and p27KIP1, two known FOXO1/3a transcriptional targets regulating the cell cycle and survival [36]. Consistent with the role of NUAK1 in the Akt signaling, NUAK1 inhibition induced the nuclear accumulation of FOXO3a (Fig. 8A, B), correlated with the induction of both p21 and p27 mRNA and protein levels (Fig. 8C–E). In contrast, NUAK1 overexpression by a TET/ON system maintained the Akt Ser-473 phosphorylation. It reduced the p21 and p27 protein levels (Fig. 8F). Similar results were observed in other cancer cell lines using the NUAK1 inhibitor (Fig. 8G, H). To confirm that NUAK1 inhibition recovers FOXO1/3a transcriptional activity, we also explored its effect on targets repressed by FOXO1/3a. We focused on FoxM1; a transcription factor implicated in several oncogenic processes [37]. NUAK1 inhibition reduced FoxM1 mRNA levels at 4 h of EGF stimulation (Fig. 8I). In summary, we determined that the NUAK1/Akt axis regulates FOXO1/3a subcellular localization and represses p21 and p27 expression but induces FoxM1 expression.

Because of the relevance of the Akt/FOXO pathway in the regulation of cell cycle, senescence, and cancer cell survival, we investigated whether NUAK1 regulates these processes under growth factor stimulation. First, we analyzed the effect of NUAK1 on cell numbers. HTH-01-015 treatment significantly decreased cell numbers under normal growth conditions (Fig. 8J) and upon EGF or insulin stimulation (Fig. 8K, L). Therefore, we explored whether NUAK1 regulates senescence or cancer cell survival. Interestingly, long-term inhibition of NUAK1 dramatically induced morphological changes, including an altered distribution of the α-tubulin and nuclear fragmentation (Additional file 1: Fig. S7). Despite morphological changes and p21 induction, NUAK1 inhibition did not induce senescence (Fig. 8M). Palbociclib, a CDK4/6 inhibitor, was used as a positive control for senescence induction (Fig. 8M). Instead, NUAK1 inhibition significantly reduced cell viability in monolayer cultures, an effect significantly stronger under EGF stimulation (Fig. 8N, O). Similarly, HTH-01-015 reduced cell viability in spheroid cultures from MDA-MB-231, U87, and DLD-1 cells (Fig. 8P), suggesting a critical role of NUAK1 in regulating cancer cell survival via Akt/FOXO1/3a axis upon growth factors stimulation and, thus, NUAK1 inhibition is a potential therapeutic approach under specific cellular contexts.

### NUAK1 inhibition potentiates pharmacological inhibition of Akt and mTOR

**Fig. 9:**
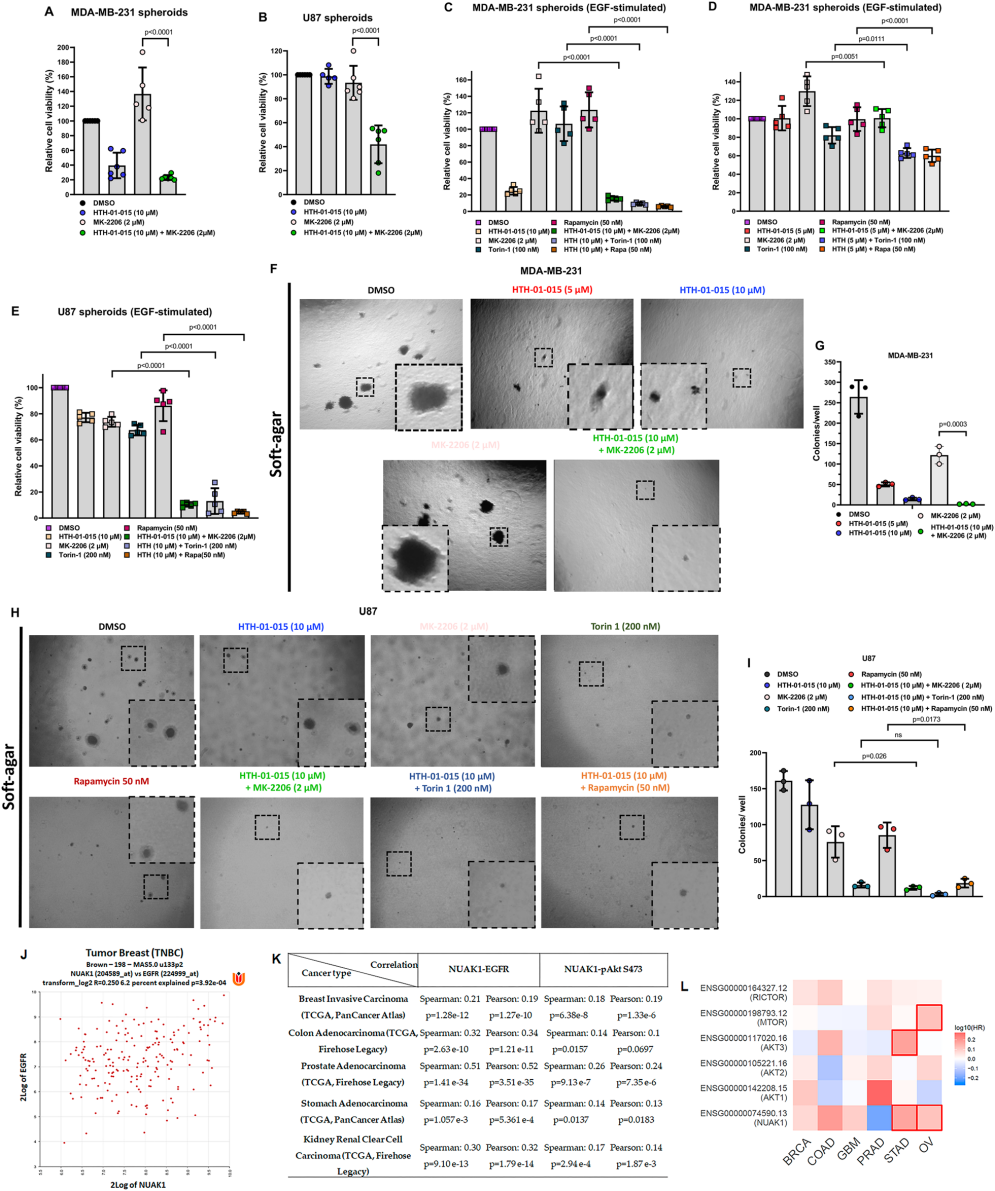
NUAK1 inhibition synergies with Akt or mTOR blockage. **A**, **B** Effect of co-targeting NUAK1 and Akt on cell viability of spheroids (3D culture) from MDA-MB-231 (**A**) and U87 (**B**) for 96 h. Cell viability measurements were described in METHODS. Each bar represents the mean ± SD, n = 5. Data were analyzed by one-way ANOVA (*P* < 0.0001 for **A**, **B**) followed by Tukey’s test (MK-2206 compared to MK-2206 plus HTH-01-015, *P* < 0.0001 for **A**, **B**). **C**, **D** Effect of co-targeting NUAK1 and Akt or mTOR on cell viability of spheroids from MDA-MB-231 cells EGF-stimulated for 48 h. HTH-01-015, 10 µM (**C**) or HTH-01-015, 5 µM (**D**). Each bar represents the mean ± SD, n = 5. Data from **C** were analyzed by one-way ANOVA (*P* < 0.0001 for Akt and NUAK1 Co-targeting; *P* < 0.0001 for mTOR and NUAK1 Co-targeting; *P* < 0.0001 for mTORC1 and NUAK1 Co-targeting) followed by Tukey’s test (MK-2206 compared to MK-2206 plus HTH-01-015, *P* < 0.0001; Torin 1 compared to Torin 1 plus HTH-01-015, *P* < 0.0001; Rapamycin compared to Rapamycin plus HTH-01-015, *P* < 0.0001). Data from **D** were analyzed by one-way ANOVA (*P* = 0.0016 for Akt and NUAK1 Co-targeting; *P* < 0.0001 for mTOR and NUAK1 Co-targeting; *P* < 0.0001 for mTORC1 and NUAK1 Co-targeting) followed by Tukey’s test (MK-2206 compared to MK-2206 plus HTH-01-015, *P* = 0.0051; Torin 1 compared to Torin1 plus HTH-01-015, *P* = 0.0111; Rapamycin compared to Rapamycin plus HTH-01-015, *P* < 0.0001). **E** Effect of co-targeting NUAK1 and Akt or mTOR on cell viability of spheroids from U87 cells EGF-stimulated by 48 h. Each bar represents the mean ± SD, n = 5. Data from **E** were analyzed by one-way ANOVA (*P* < 0.0001 for Akt and NUAK1 Co-targeting; *P* < 0.0001 for mTOR and NUAK1 Co-targeting; *P* < 0.0001 for mTORC1 and NUAK1 Co-targeting) followed by Tukey’s test (MK-2206 compared to MK-2206 plus HTH-01-015, *P* < 0.0001; Torin 1 compared to Torin 1 plus HTH-01-015, *P* < 0.0001; Rapamycin compared to Rapamycin plus HTH-01-015 (10 µM), *P* < 0.0001). **F** Soft-agar colony formation assays in MDA-MB-231 cells (Zoom ×4). **G** Quantification of number of colonies per well from **F**. Each bar represents the mean ± SD, n = 3. Data from **G** were analyzed by one-way ANOVA (*P* < 0.0001) followed by Tukey’s test (MK-2206 compared to MK-2206 plus HTH-01-015, *P* = 0.0003). **H** Soft-agar colony formation assays in U87 cells (Zoom ×4). **I** Quantification of number of colonies per well from **H**. Each bar represents the mean ± SD, n = 3. Data from **I** were analyzed by one-way ANOVA (*P* = 0.0001 for Akt and NUAK1 Co-targeting; *P* < 0.0001 for mTOR and NUAK1 Co-targeting; *P* = 0.0002 for mTORC1 and NUAK1 Co-targeting) followed by Tukey’s test (MK-2206 compared to MK-2206 plus HTH-01-015, *P* = 0.026; Torin 1 compared to Torin1 plus HTH-01-015, not significant (ns); Rapamycin compared to Rapamycin plus HTH-01-015, *P* = 0.0173). **J** Correlation between NUAK1 and EGFR expression in TNBC (Brown n = 198, MAS5.0 u133p2) from R2: Genomics Analysis and Visualization Platform. **K** Correlation between NUAK1 and EGFR expression, and NUAK1 expression and Akt Ser-473 phosphorylation in Breast Invasive carcinoma (n = 874), COAD (n = 636), Prostate Adenocarcinoma (n = 498), STAD (n = 440), and Kidney Renal Clear Cell Carcinoma (n = 537) from c-Bioportal. **L** Hazard Ratio (HR) plot for NUAK1, Akt1, Akt2, Akt3, mTOR, and Rictor in BRCA (Breast Carcinoma), COAD, GBM (Glioblastoma Multiforme), PRAD (Prostate Adenocarcinoma), STAD (Stomach Adenocarcinoma) and OV (Ovarian cancer) from Gepia2

Recently, new drugs or multi-kinase inhibitors targeting NUAK1 plus other kinases were developed [38–40], suggesting that NUAK1 is an attractive target for cancer therapy. Nevertheless, the cellular context, molecular mechanisms, and valuable drug combinations are not well-defined. The PI3K/Akt/mTOR signaling is hyperactivated in many cancers, inducing tumorigenesis and resistance to chemotherapy [8, 9]. However, a robust and durable response has not been observed [41, 42]. Therefore, combined inhibition or co-targeting emerged as an alternative drug therapy approach. Due to the new role of NUAK1 in regulating the Akt signaling and mTOR, we asked whether a combined inhibition of NUAK1 and Akt or mTOR may benefit cancer therapy. HTH-01-015, in combination with an allosteric Akt inhibitor (MK-2206), potentiated the reduction of cell viability in spheroids from MDA-MB-231 and U87 cells in basal conditions at 96 h (Fig. 9A, B). A more robust and faster effect was observed under EGF-stimulation in MDA-MB-231 and U87 spheroids at 48 h (Fig. 9C–E).

NUAK1 coordinates Akt activation, and the inhibition of mTOR induces Akt activation-dependent resistance mechanisms [43, 44]. Thus, we investigated whether a combined inhibition of NUAK1 and mTOR may be an approach to avoid Akt reactivation, causing cancer cell death. Like the combination with the Akt inhibitor, HTH-01-015 and Torin-1 (mTORC1/2 inhibitor) or rapamycin (mTORC1 inhibitor) induced a synergistic reduction in cell viability in spheroids (Fig. 9C–E). We confirmed the synergistic effect between NUAK1 and Akt inhibition in MDA-MB-231 cells (Fig. 9F, G) and between NUAK1 and Akt or mTOR inhibition in U87 cells (Fig. 9H, I) via soft-agar colonies formation assays. All these results confirmed that NUAK1 impacts cancer cell survival, indicating that its inhibition, either alone or combined with other drugs that block Akt or mTOR activity, could be a therapeutic strategy in cancers with hyperactivated Akt signaling.

### NUAK1 is associated with EGFR/Akt signaling in several types of cancer

To validate the relevance of the identified NUAK1/Akt axis, we investigated whether NUAK1 expression correlates with the expression of upstream modulators of Akt signaling and Akt phosphorylation in human cancers. Previously, high NUAK1 expression was associated with poor prognosis in glioma, ovarian cancer (OV), and non-small cell lung cancer (NSCLC) [45–47]. We analyzed TCGA data from triple-negative breast cancer (TNBC) and we found a positive correlation between NUAK1 and EGFR expression (Fig. 9J). In other cancers, NUAK1 expression also positively correlates with EGFR expression and Akt Ser-473 phosphorylation (Fig. 9K). Because NUAK1 inhibition synergies with Akt or mTOR blockage, we investigated the cancers where co-targeting may be used. Survival meta-analysis showed that NUAK1, Akt isoforms (Akt1, Akt2, and Akt3), mTOR, and Rictor positively correlate with a high hazard ratio (HR) in BRCA, COAD, GBM, PRAD, STAD, and OV (Fig. 9L), the same types of cancer where NUAK1 correlates with EGFR expression and Akt Ser-473 phosphorylation. Therefore, these analyses strongly suggest that NUAK1 is associated with the Akt activation downstream of the EGF-signaling in human cancers, supporting further studies to investigate whether targeting the NUAK1/Akt axis could be therapeutically beneficial.

## Discussion

Multiple receptor tyrosine kinases are expressed in cancer cells, mediating the activation of the PI3K/Akt signaling in tumor initiation, progression, and resistance to therapies. Therefore, there are significant efforts to identify new molecular mechanisms or signaling crosstalk involved in regulating and activating this signaling as well as new targets. Our studies identified NUAK1 as a novel kinase inducing Akt-activation and -substrate specificity downstream of the EGFR and the Insulin Receptor (IR) signaling. NUAK1 regulation and function were mainly associated with several stress conditions [12, 18], but it recently emerged with a critical role in growth factor signaling [19, 20]. We identified that NUAK1 interacts with Akt and induces Akt Ser-473 phosphorylation upon growth factor stimulation. The correlation analyses using TCGA data suggested that the NUAK1/Akt axis is conserved in several types of cancer. In a recent parallel study, KI-301670, a new NUAK1 inhibitor, showed an anti-tumor effect by directly suppressing pancreatic cancer cell growth. Like our results, KI-301670 reduced the PI3K/Akt signaling and induced p27 levels [40]. ON123300, a multi-kinase inhibitor with high specificity for CDK4/CDK6 and NUAK1, showed a strong anti-tumorigenic effect on breast cancer, glioblastoma, lymphoma, and multiple myeloma and reduced Akt phosphorylation [48–51]. However, they did not explore how KI-301670 or ON123300 impacts on the Akt phosphorylation. Together with our studies, they point to NUAK1 as a potential target for those cancers with deregulated Akt signaling. In melanomas and skin tumors, NUAK2 expression positively correlated with the Akt Ser-473 phosphorylation [52, 53]. Whether NUAK2 is also relevant for the regulation of Akt phosphorylation is unknown, but it would be essential to address it because NUAK1 and NUAK2 are differentially expressed in normal and cancer tissues.

Our studies suggest that NUAK1 and mTORC2 govern Akt-substrate specificity. NUAK1/Akt signaling regulates mainly FOXO1/3a, while mTORC2/Akt signaling regulates FOXO1/3a and TSC2 phosphorylation. Recently, we reported that NUAK1 is located in the cytoplasm and the nucleus via active nuclear transport [21]. Our current studies demonstrated that NUAK1 co-localizes with early endosomes independently of growth factor stimulation but not with late endosomes, lysosomes, or PM. The association of NUAK1 with the early endosomes is novel and may explain why NUAK1 induces the Akt-substrate specificity for FOXO1/3a but not for TSC2. In zebrafish, Appl1 regulates Akt activation and GSK3β phosphorylation, but not TSC2, at the early endosome upon growth factor stimulation [30]. In addition, endosomal mTORC2 is required for Akt-dependent FOXO1/3a and GSK3β phosphorylation in Glioblastoma cells [28, 31]. Therefore, we suggest that the subcellular distribution of the upstream modulators of Akt, including NUAK1, is critical for coordinating Akt signaling activation.

We revealed a crosstalk between the signaling led by NUAK1 and mTORC2. Compared with mTORC1, the regulation of mTORC2 and signaling crosstalk are less understood [23]. Distinct pools of mTORC2 at different subcellular locations could underlie mTORC2 signaling through other effectors [54]. NUAK1 inhibition resulted in substantial accumulation of mTOR at the lysosome and reduction of its association with Rab5 + early-endosomes. These results suggest that NUAK1 activity is necessary to maintain an appropriate subcellular distribution and activity of mTOR. Recently, new proteins have been involved in regulating the mTOR association with the lysosome, which preferentially impacts mTORC1 signaling [55, 56]. However, they did not explore an effect on mTORC2 accumulation at the lysosome or their signaling. Because NUAK1 interacts with mTORC2, we speculate that their interaction is mainly responsible for maintaining a homogeneous mTORC2 distribution and activation. Therefore, it is a subject of future studies to determine how NUAK1 interaction with mTORC2 components coordinates mTOR subcellular distribution and, consequently, the compartmentalized and full activation of Akt.

Activation of mTORC1 by nutrients correlates with its association with peripheral lysosomes close to upstream signaling proteins [57]. Additionally, peripheral clustering of the lysosomes also induces a faster reactivation of the mTORC2/Akt signaling under growth factor stimulation, suggesting a pool of Akt and mTORC2 sensitive to the lysosome positioning [3]. Therefore, the modulation of the lysosome positioning is critical to determine lysosome functions, including its role in signaling [25]. NUAK1 appears as a new regulator of lysosome homeostasis, affecting its subcellular distribution. NUAK1 inhibition induced peripheral positioning of the lysosomes without affecting the positioning of Rab5+-early endosomes. Despite their peripheral positioning, it delayed the EGF-dependent Akt activation. Although this result was unexpected, it revealed that early Akt activation requires NUAK1 activity. The effect of NUAK1 is likely due to its direct phosphorylation of Akt, supported by the kinase assays and the PLA experiments showing that NUAK1 and Akt interact within 10 min of EGF stimulation. Additionally, NUAK1 is located at the early endosomes where Akt phosphorylation drives its specificity for FOXO1/3a phosphorylation. Nevertheless, we cannot discard that NUAK1 additionally affects the Akt phosphorylation through its effect on the mTOR subcellular distribution as a mTORC2-dependent mechanism. Because endosomal mTORC2 can phosphorylate Akt, the observed reduction of mTOR association with the endosomes upon NUAK1 inhibition could contribute to the decrease in Akt phosphorylation. On the other hand, the mTORC2-dependent late activation of Akt may relate to the consequent mTOR accumulation at the lysosomes located at the cell periphery. How NUAK1 operates to control lysosomal positioning is unknown. However, according to its effect on the Akt Ser-473 phosphorylation and the mTOR subcellular distribution, our study provides evidence of a fine-tuned crosstalk between NUAK1 and mTORC2 coordinating the Akt signaling activation .

Downstream, NUAK1’s regulation of FOXO1/3a upon growth factor stimulation is novel, but a significative effect of NUAK1 on GSK3β S9 phosphorylation was not observed. Nevertheless, under normal growth conditions and oxidative stress, we observed a strong effect of NUAK1 on GSK3β S9 phosphorylation (Additional file 1: Fig. S4A, B). Previously, it was reported that NUAK1, through MYPT1 phosphorylation (S445), regulates GSK3β S9 phosphorylation under oxidative stress [18]. Therefore, NUAK1’s effect on GSK3β could depend on the cellular context, including genetic context and/or cellular conditions, such as upon growth factors stimulation or stress. Other studies showed that NUAK1 regulates cell proliferation, inducing the expression of proliferative genes and repressing anti-proliferative genes, including p21CIP1 and p27KIP1 [13]. Still, they did not determine the mechanisms involved. As mentioned above, KI-301670 also induced p27 levels [40]. Thus, our study indicates that NUAK1 through Akt/FOXO1-3a regulates p21, p27, and FoxM1 expression upon growth factor stimulation. Opposite to our results, NUAK1 induced p21 expression by phosphorylation and activation of the transcription factor p53 under glucose starvation [14]. We discard this NUAK1/p53 axis because MDA-MB-231 cells carry an inactivated p53 mutant [58]. In addition, our studies identified a mechanism by which NUAK1 may promote cancer cell survival and proliferation. Instead, the NUAK1/p53 studies indicated a mechanism for NUAK1 induction of cell cycle arrest under glucose starvation. Although the mechanism was unknown, previous studies demonstrated that NUAK1 promotes cancer cell proliferation and survival [12, 13]. We found that NUAK1 promotes cell survival in a growth factor-dependent manner, likely via Akt/FOXO1/3a. Thus, we identified a novel NUAK1/Akt/FOXO1-3a axis, which may be implicated in cancer progression and chemotherapy efficacy.

Recent evidence suggests that NUAK1 is a novel candidate for chemotherapy alone or combined with other targets [38–40, 59–61]. Based on the new mechanisms described and their apparent conservation in several types of cancer, we explored combined NUAK1 and Akt or mTOR inhibition to avoid compensatory mechanisms that result in cancer cell survival and resistance to chemotherapy. In the case of different mTOR inhibitors, the co-targeting of mTOR with upstream modulators of Akt signaling has emerged with promising results [43, 44]. Therefore, our study provides a rationale to explore the potential therapeutic outcome of NUAK1 inhibition and additional combinations with Akt or mTOR inhibitors in several types of cancer.

## Conclusions

Recently, studies described a critical role of NUAK1 in growth factor signaling via the regulation of YAP and TGF-β/SMAD pathways [19, 20]. Here, we found that NUAK1, a member of the AMPKα family, is a novel regulator of the Akt signaling. Mechanistically, NUAK1 coordinates Akt signaling upon growth factor stimulation. NUAK1 regulates mTOR location and lysosome positioning and directly phosphorylates Akt at Ser-473, and according to its subcellular localization, induces the Akt/ FOXO1-3a axis. NUAK1 promotes cancer cell survival through these mechanisms, and its inhibition potentiated Akt and mTOR pharmacological inhibition. Therefore, targeting NUAK1 or combined inhibition with Akt or mTOR inhibitors may be considered in cancer treatments.

## Materials and methods

### Antibodies

Anti-pS473 Akt (4060), Anti-pT308 Akt (4056), Anti-pan Akt (4691), Anti-NUAK1 (4458), Anti-pS2448 mTOR (2971), Anti-mTOR (2983), Anti-pS1462 TSC2 (3611), Anti-pT24/pT32 FOXO1/3a (9464), Anti-FOXO1 (L27), Anti-pS9 GSK3β (9336), Anti-GSK3β (D5C5Z), Anti-Lamp1 (9091), Anti-Raptor (2280), Anti-pT389 S6K (9206), Anti-p70 S6 Kinase (2708), Anti-pS235/S236 S6 (2211), Anti-S6 (2317), Anti-pT37/46 4EBP1 (9459), Anti-4EBP1 (9644), Anti-Myc-Tag (2276), and Anti-HA-Tag (C29F4) from Cell Signaling (Danvers, MA, USA). Anti-p21 (F-5), Anti-p27 (M-197), Anti-TSC2 (C20), Anti-mTOR (N-19), Anti-MYPT1 (C-6), Anti-GST (B14), Anti-β-actin (C-2), and Anti-GAPDH (6C5) from Santa Cruz Biotechnology (Dallas, TX, USA). RalA (05-586) from Merck Millipore (Burlington, MA, USA). Anti-Rictor (PLA0309), Anti-α-Tubulin (DM1A), Anti-FLAG (M2), Anti-HA (Clone HA-7), and Anti-Rab5 (R7904) from Sigma-Aldrich (San Luis, MO, USA). Anti-His (31212) from ThermoFisher Scientific (Waltham, MA, USA). Anti-pS445 MYPT1 (68-0043-100) from Ubiquigent (Dundee, Scotland, UK) was provided for Dr. Daniel Murphy.

### Chemicals and recombinant proteins

HTH-01-015 (SML1446-25MG), Rapamycin (R0395), GST-NUAK1 (SRP5237), GST-Akt1 (SRP5001), Insulin (I2643-25MG), H_2_O_2_ 30% (H1009), and 3XFlag-peptide (F4799) from Sigma-Aldrich. MK-2206 (11593), PD0332991 (Palbociclib) (16273), and Doxycycline (14422) from Cayman Chemical (Ann Arbor, MI, USA). WZ4003 (HY-15802) from MedChemExpress (Monmouth Junction, NJ, USA). His-NUAK1 (PV4127), Lipofectamine 3000, and Phalloidin (A12379) from ThermoFisher Scientific. Puromycin (CAS58-58-2) and A/G plus agarose (sc-2003) from Santa Cruz Biotechnology. λ-Phosphatase (P0753S) from New England BioLabs (Ipswich, MA, USA). rhEGF (78006.1) from Stemcell Technologies (Vancouver, Canada). Adenosine 5′-triphosphate, [γ-^32^P]-(NEG035C005MC) from PerkinElmer (Waltham, MA, USA). Torin-1 a gift from Drs. Nathanael S. Gray (Stanford University) and David M. Sabatini.

### Plasmids

The pCMV FLAG-hNUAK1 (DU6359) plasmid was purchased at the Medical Research Council (MRC), UK. To generate pCW57 FLAG-hNUAK1 WT, hNUAK1 WT was amplified from pCMV FLAG-hNUAK1 and subcloned into pCW57-MCS1-2 A-MCS2 using NheI and AgeI restriction sites. pCMV FLAG-hNUAK1 K84A was generated by subcloning of FLAG-hNUAK1 K84A from pBABE FLAG-hNUAK1 K84A using EcoRI restriction sites. pBABE FLAG-hNUAK1 K84A was previously generated by site direct mutagenesis using the following primers: hNUAK1_K84A_F: GGCCGAGTGGTTGCTATAGCCTCCATTCGTAAGG, hNUAK1_K84A_R: CCTTACGAATGGAGGCTATAGCAACCACTCGGCC. pCMV FLAG-NUAK1 K84A mutation was confirmed by sequencing (Additional file 1: Fig. S6). pCW57-MCS1-2 A-MCS2 (#71782), FLAG FOXO3a (#8360), pCMV dR8.2 (#8360), pCMV VSVG (#8454), pLKO scramble (#1864), pLKO shRictor (#1853), Lamp1-YFP (#2532), Lamp1-RFP (#1817), EGFR-GFP (#32751), pRK5 Myc Rictor (#11367), pRK5 Myc Raptor (#1859), and mRFP-Rab5 (#14437) from Addgene. pCMV6 Myr-Akt1-HA was kindly provided by Dr. Philip Tsichlis, The Ohio State University, Rab7-GFP by Dr. Julio Tapia, Universidad de Chile, Chile, and pINDUCER10 shNUAK1 #1 and shNUAK1 #2 by Drs. Giacomo Cossa and Martin Eilers, University of Würzburg, Germany.

### Cell culture

MDA-MB-231, HEK293T, U87 and iMEFs cells were grown in Dulbecco’s modified Eagle’s medium (DMEM) (Hyclone, Logan, UT, USA) supplemented with 10% (v/v) fetal bovine serum (FBS; Hyclone), 1% glutamine (Invitrogen, Waltham, MA, USA). DLD-1 and SW480 cells were grown in Roswell Park Memorial Institute Medium (RPMI) (Hyclone) supplemented with 10% FBS. The cell lines were regularly tested (every 4 months) for mycoplasma using EZ-PCR Mycoplasma Test Kit (Biological Industries, Beit Haemek, Israel). For lentiviral infection, HEK293T cells were transfected with 5 µg of the lentiviral vectors (pINDUCER10 shNUAK1 #1/#2, pLKO (scramble or shRictor) or pCW57 FLAG hNUAK1 WT), 5 µg of pCMV dR8.2 and 0.5 µg of pCMV VSVG. Cells were then cultured by 48 h, collecting virus-containing supernatant with 8 µg/mL of Polybrene (Sigma-Aldrich) every 24 h. For positive clone selection, we used puromycin at 2 µg/mL for 6 days.

### Western blots analysis

Proteins from cell lysates (30–50 µg) were fractionated by SDS-PAGE and transferred to PVDF membrane (Immobilon; Merck Millipore). The PVDF membranes were blocked for 1 h at room temperature in 5% nonfat milk in TBS-T and incubated with primary antibody at 4 °C overnight. After washing, the membranes were incubated for 1 h at room temperature with horseradish peroxidase-conjugated secondary antibodies diluted in TBS-T buffer. Immunolabeled proteins were visualized by ECL (RPN2209) from Cytiva (Marlborough, MA, USA).

### Immunoprecipitation

Proteins were extracted from cultured cells using lysis buffer (25 mM Tris-HCl pH 7.4, 150 mM NaCl, 0.2 mM EDTA, 1% NP40, 5% Glycerol and 2 mM MgCl_2_) with protease and phosphatase inhibitors followed by immunoprecipitation at 4 °C for 6 h, washing three times in IP wash buffer (10 mM HEPES (pH 7.9), 1.5 mM MgCl_2_, 300 mM NaCl, 10 mM KCl and 0.5% TritonX-100) and immunoblotting. For FLAG-immunoprecipitation, we used 1 µg of anti-FLAG M2 from Sigma. For HA-immunoprecipitation, we used 1 µg of anti-HA clone 7 from Sigma and protein A/G plus agarose from Santa Cruz Biotechnology.

### Mass spectrometry analysis

Multidimensional protein identification technology (MudPIT) performed in iMEF cells expressing pBABE FLAG (control), pBABE FLAG-mNUAK1 WT, or pBABE FLAG-mNUAK1 KR44/71AA (cytoplasmic mutant) were previously described in Palma et al. [21].

### In vitro kinase assay

For Akt1 phosphorylation assay, myr-Akt1-HA was expressed in HEK293T and purified via immunoprecipitation with anti-HA (Clone HA-7). Purified myr-Akt1-HA was dephosphorylated at 30 °C for 30 min. Once dephosphorylated, we performed the in vitro kinase radioactive or non-radioactive assay using His-NUAK1 (500 ng), GST-NUAK1 (500 ng) or FLAG-NUAK1 WT and FLAG-NUAK1 K84A. FLAG-NUAK1 WT and FLAG-NUAK1 K84A (kinase-dead) were purified by immunoprecipitation (IP) from HEK293T and then eluted from the resin using 3XFLAG-peptide. All the reactions were incubated at 30 °C for 60 min using the buffer kinase non-radioactive (50 mM Tris-HCl (pH 7.5), 100 mM KCl, 50 mM MgCl_2_, 1 mM Na_3_VO_4_, 1 mM DTT, 5% glycerol, and 0.5 mM ATP) or buffer kinase radioactive [50 mM Tris-HCl (pH 7.5), 100 mM KCl, 50 mM MgCl_2_, 1 mM Na_3_VO_4_, 1 mM DTT, 5% glycerol, 0.25 mM ATP, and 10 µCi [γ-^32^P] ATP]. For NUAK1 and Akt1 phosphorylation assay, GST-Akt1 (500 ng) and His-NUAK1 (500 ng) were combined in buffer kinase assay (non-radioactive) and incubated at 30 °C for 60 min. All samples were denatured for 3 min at 100 °C and analyzed by Western blot or autoradiography.

### Confocal fluorescence microscopy

Cells plated on coverslips were fixed (4% paraformaldehyde), permeabilized (0.1% Triton X-100) and incubated with corresponding primary antibody overnight (3% BSA in PBS). After washing, fixed cells were incubated with the corresponding Alexa Fluor coupled secondary antibody, Hoechst 33342 and Phalloidin for 2 h. Proximity Ligation Assays (PLA) were carried out using the Duolink In Situ Red started Kit Mouse/Rabbit (DUO92101, Sigma-Aldrich) according to the manufacturer’s protocol. Images were obtained with LMS780 spectral confocal system (Zeiss, Germany). Identical exposure times and zoom (63× for IF and 40× for PLA) were used for comparison and quantification.

### qPCR

For gene expression analysis, total RNA was extracted from cells with Trizol reagent (Invitrogen; 15596026). Real-time PCR was performed using KAPA SYBR FAST qPCR Master Mix (2X) Kit (Sigma-Aldrich; KK4601) and the AriaMX Real-Time PCR System (Agilent) according to the manufacturer’s instructions. The qPCR primers used are h_p21_F: GGCAGACCAGCATGACAGAT, h_p21_R: AGATGTAGAGCGGGCCTTTG, h_P27_F: GCAAGTACGAGTGGCAAGAG, h_p27_R: CCAAATGCGTGTCCTCAGAG, h_FOXM1_F: GCAGGCTGCACTATCAACAA, h_FOXM1_R: TCGAAGGCTCCTCAACCTTA. β-Actin (*ACTB*) amplification was used as reference gene.

### Senescence, cell death, and soft-agar assays

For senescence, we used Senescence β-Galactosidase Staining Kit (9860, Cell Signaling Technology) following the manufacturer protocol. For cell death assays in 2D culture, we used IncuCyte. Two thousand cells were seeded for each case in 96 well plates and cell death was monitored at each hour using SYTOX (ThermoFisher Scientific). For cell viability assays in 3D culture, spheroids were generated in 96 Ultra-Low Attachment plates by 4 days. Cell viability was measured using CellTiter-Glo® 3D Cell Viability Assay (G9683, Promega) following manufacturing instructions. For soft-agar assays, 2500 MDA-MB-231 cells per 12-well plate or 1250 MDA-MB-231 or U87 cells in 24-well plate were seeded in 1.8% of Bacto-agar in DMEM supplemented with 10% FBS. Every 4 days the cells were resupplied with fresh media. After 3 weeks of incubation the colonies were counted.

### Bioinformatical analysis

Correlation analyses were performed at https://hgserver1.amc.nl/ and https://www.cbioportal.org/. Kaplan–Meier curves were obtained at https://kmplot.com/analysis/ and http://gepia2.cancer-pku.cn/. Molecular docking was performed in Hex 8.0 docking software and visualized in ChimeraX 1.1. Putative phosphorylation sites were identified using GPS 5.0 http://gps.biocuckoo.cn/online.php.

### Quantification and statistical analysis

Densitometry quantification and co-localization analyses were performed in ImageJ/FiJi software. The Immunofluorescence quantifications were performed in ImageJ measuring fluorescence intensity followed by normalization with their respective control. The analysis of lysosome distribution was performed by measuring the fractional distance of the lysosome from the nucleus. Briefly, a boundary was drawn along the periphery of each selected cell using a freehand selection tool. The Lamp1 signal from nearby cells was removed using the clear outside function of Fiji software. Next, a ROI was drawn around the nucleus (Hoechst signal), and Lamp1 fluorescent intensity was measured (First ROI). Then, Lamp1 intensity was measured for each ROI incremented by 5 μm until the cell periphery. Finally, Lamp1 intensity was calculated for perinuclear (0–5 μm) by subtracting the intensity of the first ROI (nucleus) with the second, and peripheral (> 10 μm or > 15 μm) by subtracting the intensity of the third (> 10 μm) or fourth (> 15 μm) ROI with the total cell intensity. Statistical analysis and graphics were performed with *GraphPad Prism* 9.

### Supplementary Information

**Additional file 1: Figure S1.** NUAK1 inhibition does not impact on mTOR-Rictor association. **Figure S2.** NUAK1 effect on mTOR subcellular distribution. **Figure S3.** NUAK1 inhibition does not affect early endosomes distribution. **Figure S4.** NUAK1 effect on Akt signaling under different cellular conditions. **Figure S5.** NUAK1 co-localize with endogenous Rab5. **Figure S6.** Validation of NUAK1 Kinase Dead mutant (K84A). **Figure S7.** Long-term inhibition of NUAK1 dramatically induces morphological changes.

## Data Availability

The data and material that support the findings of this study are available upon request to the corresponding authors.

## Abbreviations

AMPK: Adenosine 5′-monophosphate-activated protein kinase
EGFR: Epidermal growth factor receptor
FOXO: Forkhead box O
GSK3: Glycogen synthase kinase 3
iMEF: Immortalized Mouse Embryonic Fibroblast
IR: Insulin Receptor
ishRNA: Inducible small hairpin RNAs
LKB1: Liver Kinase B1
mTOR: Mechanistic target of rapamycin
MudPIT: Multidimensional Protein Identification Technology
NSCLC: Non-small cell lung cancer
PI3K: Phosphatidylinositol 3 kinase
PLA: Proximity Ligation Assay
PM: Plasma membrane
Raptor: Regulatory-Associated Protein of Mammalian Target of Rapamycin
Rictor: Rapamycin-Insensitive Companion of mTOR
TNBC: Triple negative breast cancer
TSC2: Tuberous sclerosis complex 2
