# Supplementary material for: NUAK1 coordinates growth factor-dependent activation of mTORC2 and Akt signaling
Source: Cell Biosci. 2023 Dec 22;13:232. doi: 10.1186/s13578-023-01185-2 (PMC10740258; doi:10.1186/s13578-023-01185-2)
Supplement: Supplementary file 1 — Additional file 1: Figure S1. NUAK1 inhibition does not impact on mTOR-Rictor association. Figure S2. NUAK1 effect on mTOR subcellular distribution. Figure S3. NUAK1 inhibition does not affect early endosomes distribution. Figure S4. NUAK1 effect on Akt signaling under different cellular conditions. Figure S5. NUAK1 co-localize with endogenous Rab5. Figure S6. Validation of NUAK1 Kinase Dead mutant (K84A). Figure S7. Long-term inhibition of NUAK1 dramatically induces morphological changes. [file 13578_2023_1185_MOESM1_ESM.pdf]

NUAK1 coordinates growth factor-dependent activation of mTORC2 and Akt signaling.

Mario Palma[1*], Elizabeth Riffo[1], Alejandro Farias[1], Viviana Coliboro-Dannich[1], Luis Espinoza-Francine[1], Emilia Escalona[1], Roberto Amigo[2], José L. Gutiérrez[2], Roxana Pincheira[1] and Ariel F. Castro[1*].

[1]Laboratorio de Transducción de Señales y Cáncer. Departamento de Bioquímica y Biología Molecular. Facultad Cs. Biológicas. Universidad de Concepción, Chile.

[2]Laboratorio de Regulación Transcripcional. Departamento de Bioquímica y Biología Molecular. Facultad Cs. Biológicas. Universidad de Concepción, Chile.

*Correspondence: Mario Palma: mpalma@hsph.harvard.edu; Ariel F. Castro: arcastro@udec.cl

Supplementary Information

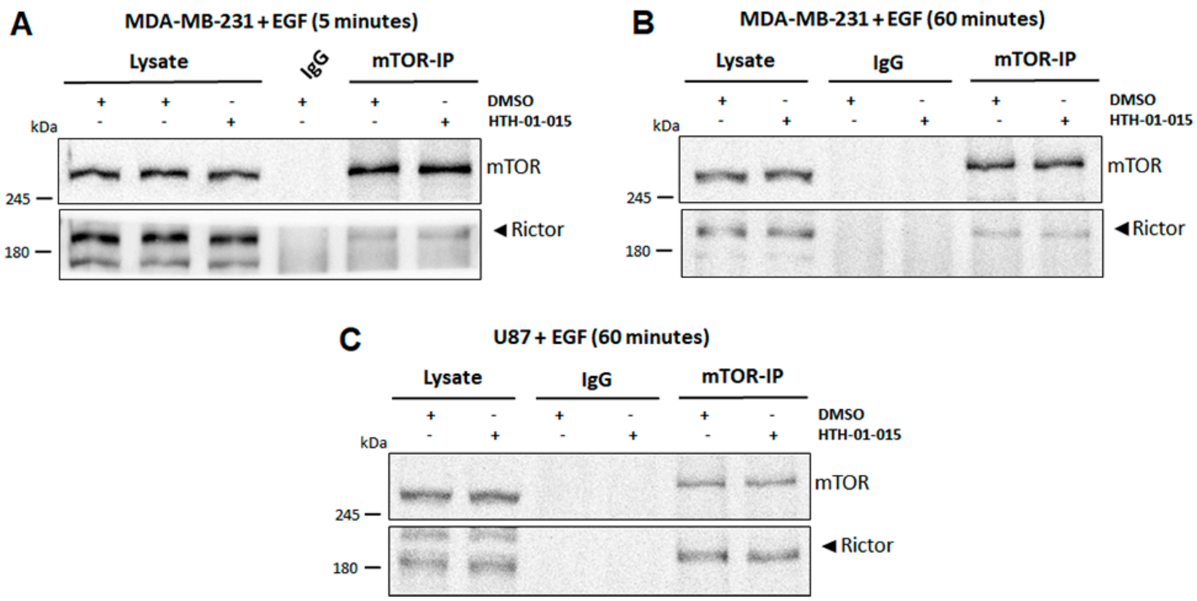

Figure S1. NUAK1 inhibition does not impact on mTOR-Rictor association.

A. Immunoblot (IB) of the Immunoprecipitation (IP) of endogenous mTOR and Co-IP of endogenous Rictor after 5 minutes of EGF stimulation in MDA-MB-231 cells. **B-C.** IB of IP of endogenous mTOR and Co-IP of endogenous Rictor after 60 minutes of EGF stimulation in MDA-MB-231 (B) and U87 (C) cells.

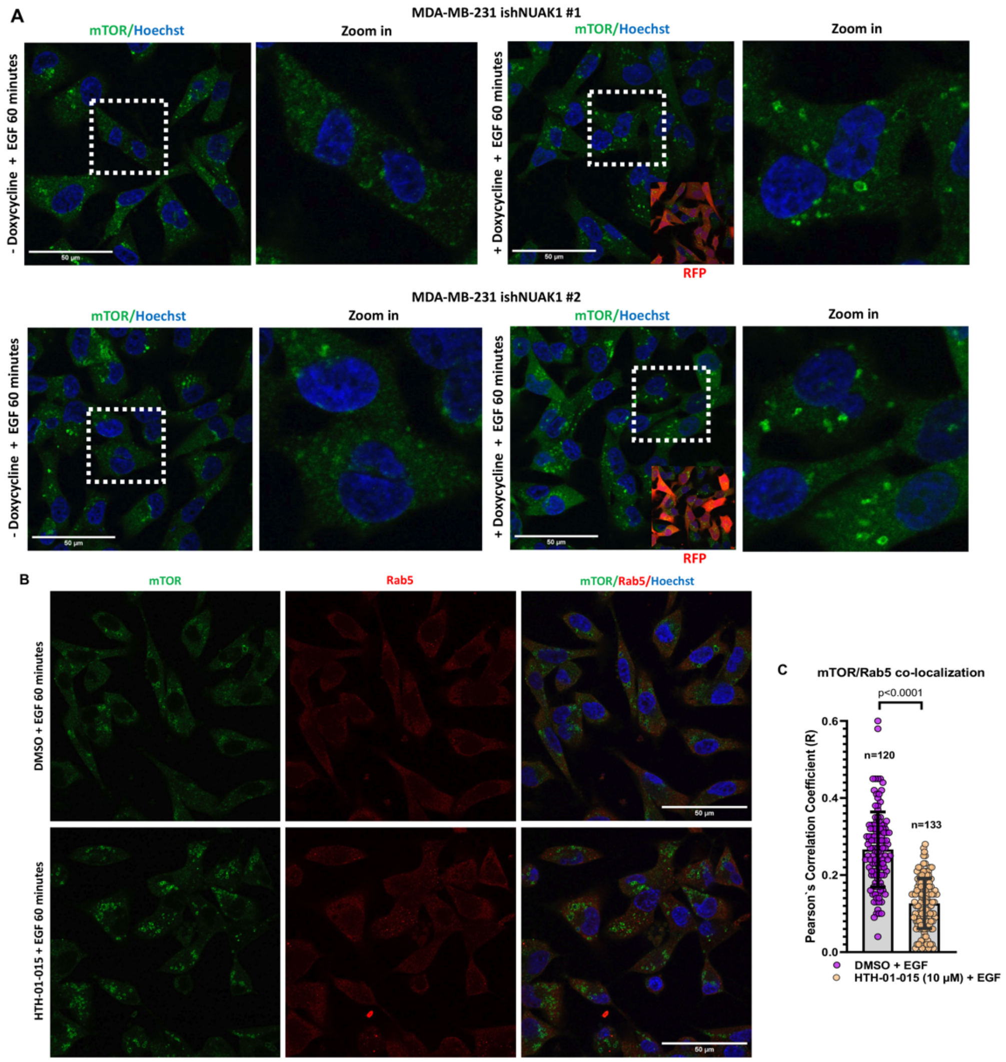

Figure S2. NUAK1 effect on mTOR subcellular distribution.

A. Representative confocal images of mTOR in MDA-MB-231 cells expressing inducible shRNAs for NUAK1. Inducible models were treated with or without doxycycline by 24 hours and then serum-starved overnight followed by stimulation with EGF for 60 minutes Green, mTOR; Red, RFP; Blue, Nuclei. **B.** Representative confocal images of mTOR and Rab5 in MDA-MB-231 cells serum-starved overnight followed by 90 minutes of pretreatment with DMSO or HTH-01-015 (10 µM) before stimulation with EGF for 60 minutes. Red, Rab5; Green, mTOR; Blue, Nuclei. **C.** Quantification of mTOR/Rab5 co-localization from B. Each bar represents the mean ± SD, Student t test.

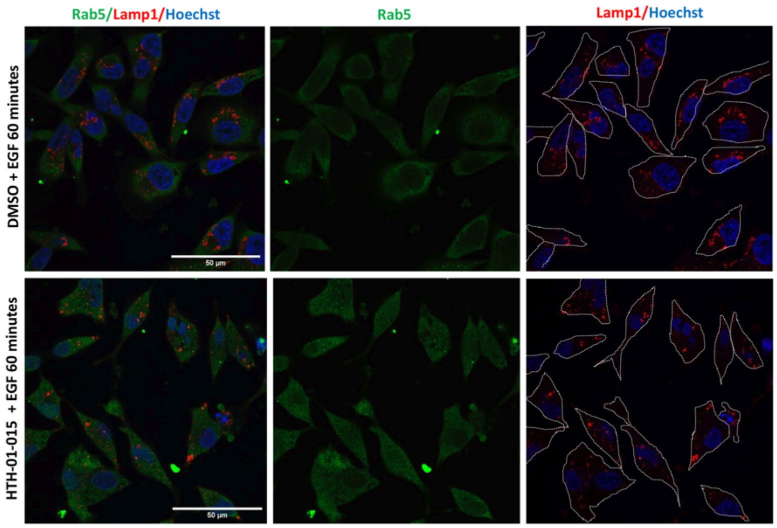

Figure S3. NUAK1 inhibition does not affect early endosomes distribution.

Representative confocal images of Rab5 and Lamp1 under NUAK1 inhibition in MDA-MB-231 after EGF stimulation for 60 minutes. Left, merge; Right, Rab5 and Lamp1 + Hoechst images. Cells borders were marked with a boundary. Green, Rab5; Red, Lamp1; Blue, nuclei

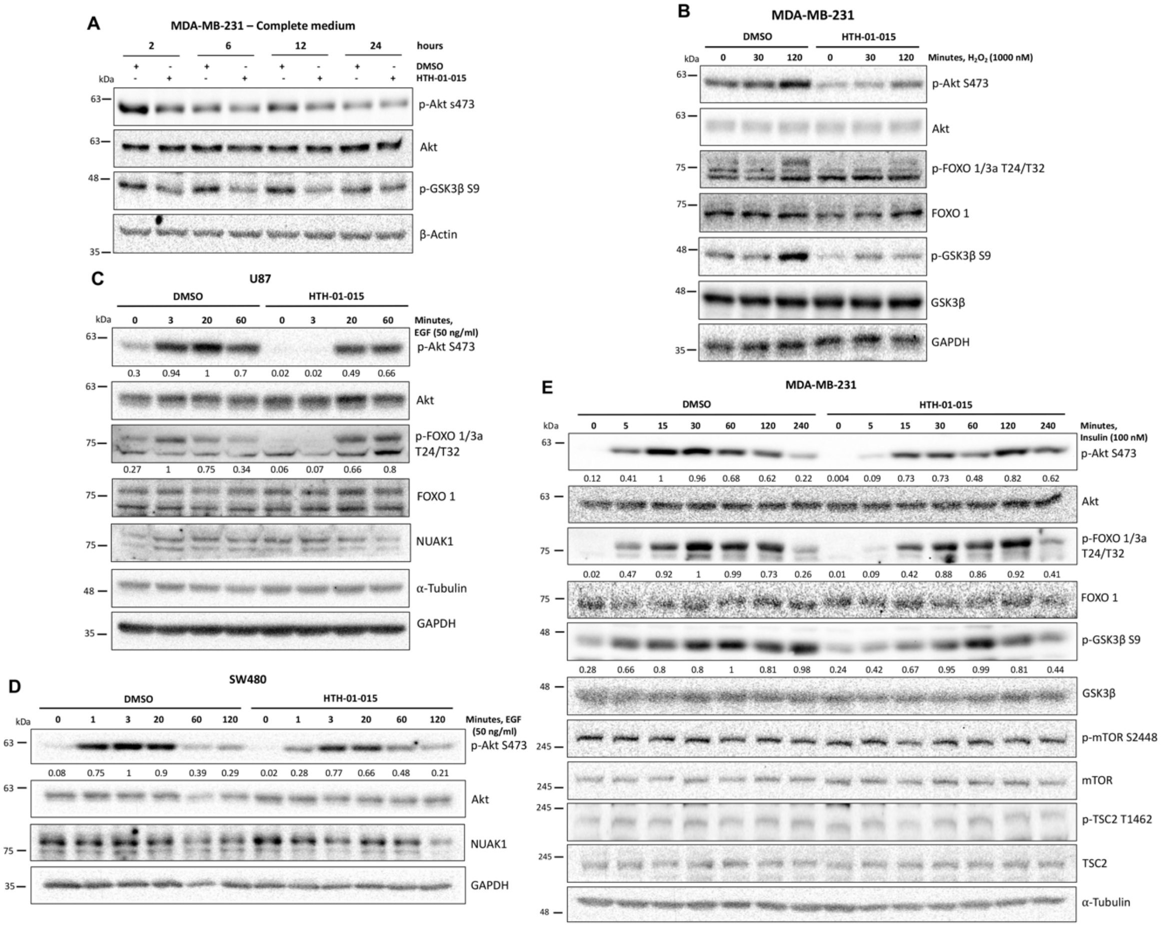

Figure S4. NUAK1 effect on Akt signaling under different cellular conditions.

A. IB of the effect of NUAK1 inhibition on Akt signaling in normal growth conditions. MDA-MB-231 cells were treated with DMSO or HTH-01-015 (10 µM) for 2, 6, 12 and 24 hours**.** β-actin was used as loading control. **B.** IB of the effect of NUAK1 inhibition on the Akt signaling upon oxidative stress. MDA-MB-231 cells were pretreated with DMSO or HTH-01-015 (10 µM) by 1-hour before treatment with H_2O_2 by 0, 30 and 120 minutes. GAPDH was used as loading control. **C-D.** IB of Akt signaling under NUAK1 inhibition in U87 (C) and SW480 (D) cells serum-starved overnight followed by 1-hour of pretreatment with DMSO or HTH-01-015 (10 µM) before stimulation with EGF. **E.** IB of Akt signaling under NUAK1 inhibition in MDA-MB-231 cells serum-starved overnight followed by 1-hour of pretreatment with DMSO or HTH-01-015 (10 µM) before stimulation with Insulin.

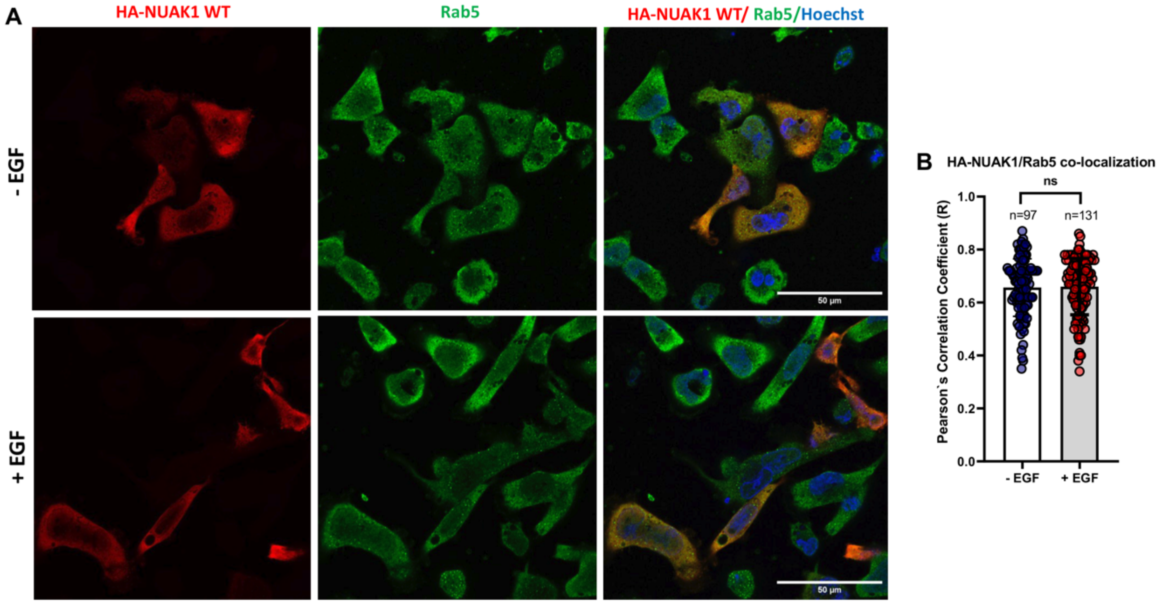

Figure S5. NUAK1 co-localize with endogenous Rab5.

A. IF of MDA-MB-231 cells after 0 (-EGF) and 15 (+EGF) minutes of EGF stimulation. Representative confocal images for HA-NUAK1 WT and endogenous Rab5. Red, HA-Tagged-NUAK1; Green, endogenous Rab5 (early-endosome marker); Blue, nuclei. **B.** Quantification of HA-NUAK1/Rab5 co-localization from A. Each bar represents the mean ± SD, not significant (ns), Student t test.

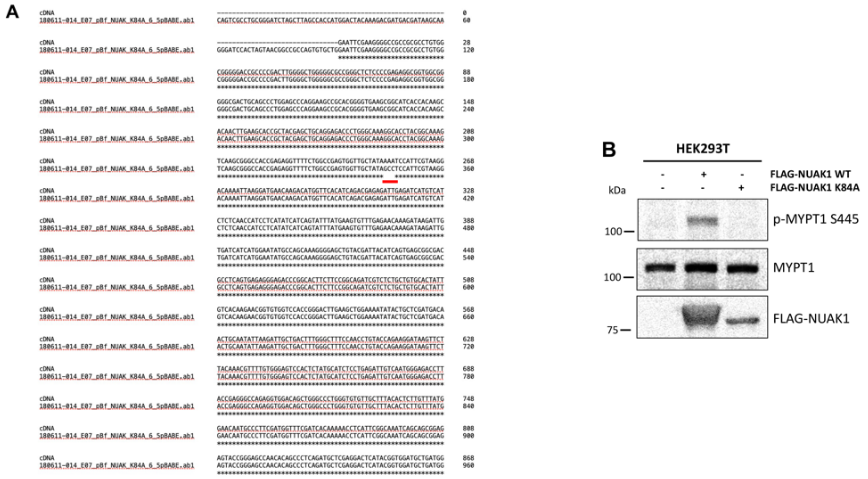

Figure S6. Validation of NUAK1 Kinase Dead mutant (K84A).

A. Alignment of human NUAK1 WT (cDNA) with the sequence obtained by sequencing of human NUAK1 K84A. The Alignment validated the mutation at Lysine 84 (K84) by Alanine (A). **B.** IB of the activity of FLAG-NUAK1 WT and FLAG-NUAK1 K84A. phospho-MYPT1 at Ser-445 was used as a positive control. NUAK1 K84A mutant does not induced MYTP1 phosphorylation.

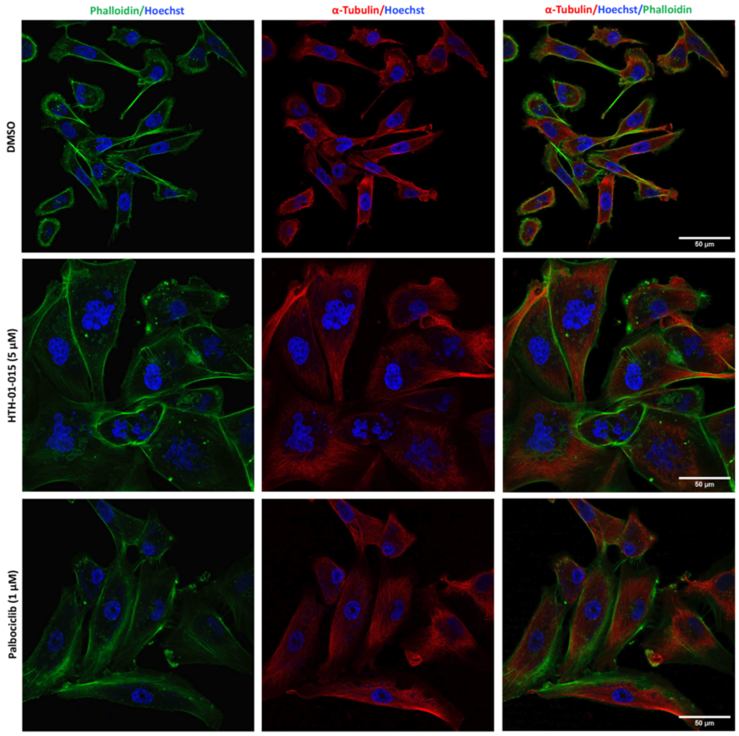

Figure S7. Long-term inhibition of NUAK1 dramatically induces morphological changes.

Representative confocal images of MDA-MB-231 cells treated with DMSO, HTH-01-015 (5 µM) or Palbociclib (1 µM) (used as a positive control) for 4 days. Red, α-tubulin; Green, Phalloidin (F-actin); Blue, nuclei (n=3).
